# Interplay of *Mycobacterium abscessus* and *Pseudomonas aeruginosa* in experimental models of coinfection: Biofilm dynamics and host immune response

**DOI:** 10.1080/21505594.2025.2493221

**Published:** 2025-04-16

**Authors:** Víctor Campo-Pérez, Esther Julián, Eduard Torrents

**Affiliations:** a Bacterial Infections and Antimicrobial Therapy Group, Institute for Bioengineering of Catalonia (IBEC), The Barcelona Institute of Science and Technology (BIST), Barcelona, Spain; b Departament de Genètica i de Microbiologia, Facultat de Biociències, Universitat Autònoma de Barcelona, Barcelona, Spain; c Microbiology Section, Department of Genetics, Microbiology and Statistics, Faculty of Biology, University of Barcelona, Barcelona, Spain

**Keywords:** Dual-species biofilm, *Galleria mellonella*, nontuberculous mycobacteria, pulmonary epithelia, immunosuppression

## Abstract

The incidence of infection by nontuberculous mycobacteria, mainly *Mycobacterium abscessus*, is increasing in patients with cystic fibrosis and other chronic pulmonary diseases, leading to an accelerated lung function decline. In most cases, *M. abscessus* coinfects *Pseudomonas aeruginosa*, the most common pathogen in these conditions. However, how these two bacterial species interact during infection remains poorly understood. This study explored their behaviour in three relevant pathogenic settings: dual-species biofilm development using a recently developed method to monitor individual species in dual-species biofilms, coinfection in bronchial epithelial cells, and *in vivo* coinfection in the *Galleria mellonella* model. The results demonstrated that both species form stable mixed biofilms and reciprocally inhibit single-biofilm progression. Coinfections in bronchial epithelial cells significantly decreased cell viability, whereas in *G. mellonella*, coinfections induced lower survival rates than individual infections. Analysis of the immune response triggered by each bacterium in bronchial epithelial cell assays and *G. mellonella* larvae revealed that *P. aeruginosa* induces the overexpression of proinflammatory and melanization cascade responses, respectively. In contrast, *M. abscessus* and *P. aeruginosa* coinfection significantly inhibited the immune response in both models, resulting in worse consequences for the host than those generated by a single *P. aeruginosa* infection. Overall, this study highlights the novel role of *M. abscessus* in suppressing immune responses during coinfection with *P. aeruginosa*, emphasizing the clinical implications for the management of cystic fibrosis and other pulmonary diseases. Understanding these interactions could inform the development of new therapeutic strategies to mitigate the severity of coinfections in vulnerable patients.

## Introduction

Cystic fibrosis (CF) is an autosomal recessive inherited condition caused by mutations in the gene encoding cystic fibrosis transmembrane conductance regulator (CFTR) [1]. This protein acts as a transporter channel that conducts chloride ions (Cl^−^) across epithelial cell membranes to maintain the balance between salt and water on many surfaces in the body and is especially relevant in the respiratory system airways [2]. A malfunction in the CFTR protein prevents proper hydration of the cellular surface, which leads to the loss of mucus covering the cells, which becomes thick and sticky [3]. This altered mucus impairs the primary innate defence mechanism of the lung, particularly mucociliary clearance [4]. Consequently, CF patients face an increased risk of infections from pathogens such as *Pseudomonas aeruginosa*, *Staphylococcus aureus*, *Burkholderia spp*., and nontuberculous *Mycobacterium spp*. (NTM). These infections are associated with a high risk of pulmonary compromise and frequently result in chronic infections [5]. In fact, respiratory tract infections, mainly in the lungs, of CF patients as well as other infection associated pulmonary diseases, are complex and polymicrobial, capable of developing intermicrobial and host–pathogen interactions that impact clinically relevant features such as virulence, persistent colonization of the airways, and antimicrobial recalcitrance [6,7]. Polymicrobial infections represent a significant challenge in various clinical contexts, extending far beyond CF as chronic wounds, odontogenic infections, chronic otitis, etc. These infections involve interactions between different microbial species that can profoundly influence disease progression, host immune responses, and treatment outcomes. These interactions often result in enhanced pathogenicity, antimicrobial resistance, and immune evasion. This study highlights the importance of understanding the interplay between microbial species commonly associated with pathogenic contexts to unravel the dynamics of microbial coexistence and discern clinically relevant aspects. Although CF polymicrobial infections are highly individualized in each patient, *S. aureus* and *P. aeruginosa* are dominant in the microbial communities of children and adults, respectively. In this sense, coinfection by these two dominant pathogens or one of them with other species negatively affects respiratory function compared with single infections [7].

*P. aeruginosa* is a ubiquitous gram-negative opportunistic pathogen that is metabolically flexible and is considered the most relevant bacterium associated with CF owing to its high prevalence and pathogenesis [8]. Owing to its ability to adapt to the CF pulmonary environment, *P. aeruginosa* is also the primary pathogen present in advanced CF lung disease [9]. *P. aeruginosa* PAO1 is the reference laboratory strain for research, and PAET1 is a clinical isolate from a patient with chronic cystic fibrosis. *P. aeruginosa* develops biofilms on thick and dehydrated mucoid surfaces, which represent a difficult barrier to antibiotic penetration and host innate immune effectors in CF [10]. These biofilms cause chronic infections through increased antibiotic tolerance, resistance to phagocytosis, and immune-mediated chronic inflammation [11]. Furthermore, biofilms are usually polymicrobial and are related to an exacerbated inflammatory immune response that seriously compromises respiratory function and produces extensive lung lesions [12].

A significant increase in the incidence of infections caused by NTM in CF patients has recently been reported [13,14], with fast-growing *Mycobacterium abscessus* being the most prominent and worrisome pathogen of this genus [15]. *M. abscessus* infections increase morbidity and mortality due to prolonged treatments, significant side effects, antimicrobial resistance, and low respiratory clearance success rates [16]. In clinical studies, *M. abscessus* infection has been associated with a greater decline in lung function compared to other non-mycobacterial pulmonary infections (−2.52% vs. −1.64% predicted forced expiratory volume per year, *p* < 0.05) [15]. Additionally, it has shown the highest cumulative mortality rate among NTM, reaching 50.6% over 15 years [17]. Another relevant element in *M. abscessus* pathogenicity is its ability to modify its cell wall, which allows for the identification of two different morphotypes in this species: smooth (S), which contains abundant glycopeptidolipids (GPLs) in the outer layer, and rough (R), which is devoid of GPLs [18]. Similar to *P. aeruginosa*, *M. abscessus* also has biofilm-forming capacity, making treatment even more difficult [19]. Remarkably, 58–78% of patients with *M. abscessus* infection are also infected with *P. aeruginosa* [20]. Colonization of the lung epithelium begins with S morphotypes, which progressively switch to R mutants, resulting in a more aggressive, invasive pulmonary infection [21]. *M. abscessus* biofilms exhibit distinct mechanical properties, with R biofilms being more rigid than S biofilms, and both R/S biofilms showing greater resistance to mechanical lung clearance than *P. aeruginosa* biofilms [22]. Furthermore, *M. abscessus* biofilm bacilli change mycolic acid proportions and produce a lipid-relevant extracellular matrix with other components, such as carbohydrates, proteins, and extracellular DNA [23].

Coinfections of *P. aeruginosa* and *M. abscessus* in CF have become clinically more relevant over the last two decades, and the incidence of NTM infections among CF patients has increased from 3.3% to 22.6%, resulting in increased morbidity and mortality [18]. The *M. abscessus* complex is present in 16–68% of NTM-positive sputum cultures, and its prevalence is increasing [13]. Furthermore, the presence of *M. abscessus* lung disease has been considered a strong relative contraindication to lung transplantation [24], which can extend and improve the quality of life of patients with CF, increasing their mean life expectancy by 7.4 years [25].

Furthermore, apart from the biofilm-mediated immune evasion, *P. aeruginosa* and *M. abscessus* employ distinct mechanisms to influence host immunity, which are particularly significant during coinfections. *P. aeruginosa* utilizes quorum sensing systems to coordinate the production of virulence factors, including exotoxins and proteases, which can degrade host immune components [26]. Also, bacterial surface components, such as the lipopolysaccharide and different secretion systems, interact with the host’s immunity. *M. abscessus* have specific surface lipids, membrane proteins and ESX secretion systems, which also affect host immune responses [27]. Coinfection of both pathogens affects host immunity, creating a permissive environment for persistent infection and exacerbated tissue damage.

Despite the challenges stated above, studies focusing on the interactions between these species are scarce. It is therefore crucial to further elucidate and characterize the *P. aeruginosa-M. abscessus* coinfections. For this reason, the objective of the present study was to unravel the interaction mechanisms between both bacteria, focusing on relevant aspects of pathogenicity: dual-species biofilm development using recently developed coculture techniques, including S and R *M. abscessus* morphotypes, and the viability outcome and analysis of the triggered immune response by each species in individual and coinfection experiments using *in vitro* and *in vivo* models. This study is among the first to demonstrate immune suppression in dual-species infections involving *M. abscessus* and *P. aeruginosa*. The findings reveal that *M. abscessus* significantly dampens host immune responses during coinfection, exacerbating disease outcomes. In this regard, the study encourages the exploration of more efficient treatment strategies focused on solving specific coinfection features; for example, combinations of antibiotics with immune-modulating drugs could be explored to strengthen host defences. The findings achieved have broader implications beyond cystic fibrosis, but also other diseases associated with polymicrobial infections.

## Materials and methods

### Bacterial strains and culture conditions

Two different *P. aeruginosa* strains were used: PAO1 (ATCC 15692) as a reference laboratory strain, and PAET1, a clinical isolate from a recurrent infection chronic CF patient [28]. The PAO1 strain results from a spontaneous mutation that conferred chloramphenicol resistance of the original PAO isolate obtained in 1954 from a wound. Furthermore, the PAO1 strain has high intrinsic antibiotic resistance to ampicillin, first-generation cephalosporins and low-level resistance to some macrolides (as erythromycin) [29]. The PAET1 strain presents a similar antibiotic resistance pattern as PAO1 (data not shown). PAO1 and PAET1 strains were transformed with a plasmid (pETS130*lux*) expressing the constitutive bacterial luciferase gene cassette (*lux*) as a bioluminescent bioreporter system [30]. Both *P. aeruginosa* strains were grown in Luria-Bertani (LB, Sharlab, Barcelona, Spain) agar with 50 μg/ml gentamycin (Gm) for PAO1 and 300 μg/ml carbenicillin (Cb) for PAET1 as a selection marker. For biofilm formation assays, *P. aeruginosa* was grown in tryptic soy broth (TSB, Sharlab) without antibiotics. *Bacillus thuringiensis* (ATCC 10792) was cultured in tryptic soy agar (TSA, Sharlab) or TSB, and *Escherichia coli* MG1655 (ATCC 700926) was grown on LB (agar and broth). The *M. abscessus* DSMZ 44196 (ATCC 19977) original smooth (S) morphotype and the rough (R) variant, a natural mutant of the wild-type strain previously obtained [31], were grown on TSB with agitation and 0.5 mm diameter glass beads (DDBiolab, Barcelona, Spain) to prevent cell aggregation, and on TSA agar for colony forming unit (CFU) counting. As previously reported, both variants of *M. abscessus* were transformed with plasmid pETS218 [32]. Further details regarding the strains used are provided Supplementary Table S1.

### DNA manipulation and plasmid construction

Molecular biology enzymes and kits were purchased from Thermo Fisher Scientific (Madrid, Spain) and were used according to the manufacturer’s instructions. DNA amplification was performed by PCR using DreamTaq MasterMix (2X) or High-Fidelity PCR Enzyme Mix (Thermo Fisher Scientific). The primers used are listed in Supplementary Table S2. Plasmid pETS218 was constructed by cloning the promoter region of class Ib ribonucleotide reductase (encoded by the *nrdHIE* genes) from *M. brumae* (GenBank assembly accession: GCA_900073015.1). All other recombinant DNA manipulations were performed using the standard procedures [33]. For the construction of mycobacterial *nrdH* transcriptional green fluorescent protein (GFP) fusions, a 537 bp fragment encompassing the *nrdH* promoter region was amplified by PCR using the primer pair MbruPnrdHIE-For-MbruPnrdHIE-Rev and *M. brumae* genomic DNA; the obtained DNA fragment was cloned into pJET1.2 and transformed into *E. coli* DH5α cells. *BamHI* and *Apa*I restriction enzymes were used for fragment digestion and cloning into pFPV27 [34] to generate pETS218. The absence of mutations introduced during cloning was verified by DNA sequencing. *M. abscessus* S and R morphotypes were grown in TSA plus 50 μg/ml kanamycin (Kn) when they were transformed with pETS218, as previously described [32].

### Biofilm progression inhibition quantification

Biofilm quantification was performed using a microtiter plate screening assay that was previously optimized and validated by our group [35] and by other authors [36,37] (Supplementary Figure S1). Briefly, mature *P. aeruginosa* PAO1 and PAET1 biofilms (72 h growth) were cocultured with *B. thuringiensis*, *E. coli* and *M. abscessus* R and S for 24 h. Overnight (ON) cultures of *B. thuringiensis* and *E. coli* were adjusted to an optical density (OD_550 nm_) of 1 and appropriately diluted to obtain the desired concentrations corresponding with 10^4^ /10^5^ CFU/well. *M. abscessus* R and S were grown for three days on TSB cultures, and the concentrations of both live and heat-killed (121°C for 21 min) cell suspensions were determined by comparison with the MacFarland 1 (McF) turbidity standard. Bacterial culture supernatants were obtained by centrifugation of these cultures at 12,000 × g, and 50 µL was added per well. Serial dilutions of the bacterial suspensions were plated to verify the accurate concentration in each experiment for all bacteria tested. To assess the inhibition or stimulation of *P. aeruginosa* biofilm growth, *lux* intensity (*P. aeruginosa* cells) was quantified using a Spark multimode microplate reader (Tecan, Männedorf, Switzerland). Biofilm growth percentages were calculated for the coculture conditions by comparing the *lux* intensity to that of a monoculture *P. aeruginosa* biofilm grown under identical conditions, which was set as the 100% biofilm growth reference. For the simultaneous dual-species biofilm formation experiments, *P. aeruginosa* PAO1 and PAET1 suspensions were mixed with the remaining bacteria or supernatants and grown for 72 h.

### *M. abscessus* biofilm formation and *P. aeruginosa coexistence*

Three-day-old liquid cultures of *M. abscessus* R and S transformed with pETS218 plasmid were adjusted to a concentration of 5 × 10^6^ CFU/ml and grown in TSB medium + 0.2% glucose in Costar® 96-Well Black Polystyrene Plates (200 μl/well) (Corning) at 37°C under saturated humidity conditions to allow mycobacterial biofilm formation. After 120 h of incubation, three washes with PBS were carried out, and suspensions of *P. aeruginosa* PAO1 and PAET1 cultures were added at an OD_550 nm_ of 0.1 for an additional 72 h. Finally, the biofilms were homogenized in PBS and quantified by measuring the GFP expression associated with *M. abscessus* growth using a Spark® multimode microplate reader. Similarly to the previous section, the percentages of GFP intensity (from *M. abscessus* cells) obtained under coculture conditions were compared to individual *M. abscessus* biofilms, which were considered as 100% growth, to quantify the effect of *P. aeruginosa* on *M. abscessus* mature biofilms.

### Biofilm analysis through confocal microscopy

*P. aeruginosa* and mycobacterial biofilms that developed in 96-well plates were collected, and bacteria were stained with 4,’6-diamidino-2-phenylindole (DAPI). *M. abscessus* R and S (transformed with pETS218) bacilli were specifically detected by measuring the GFP expression. Representative images were obtained using a Zeiss LSM 800 confocal laser scanning microscope (CSLM, Zeiss, Oberkochen, Germany) with a 63×/1.4 oil objective with excitation wavelengths of 405 and 488 nm for DAPI and GFP, respectively. The structure and disposition of the bacteria in the biofilm were analyzed using orthogonal projections (Supplementary Figure S2). Five representative Z-stack images were selected and analyzed for pixel color quantification using the color histogram tool from the Fiji ImageJ software and Zen software (Zeiss).

### *Galleria mellonella* maintenance and infection

*G. mellonella* larvae were fed *at libitum* with an artificial diet, as previously described [30]. The larvae were kept at 34°C in the dark until they reached their optimum size (approximately 200 mg). *G. mellonella* larvae were injected with 10 µL of bacterial suspension at the desired concentration through the top right proleg using a 25 µl Hamilton microsyringe (Hamilton, Reno, USA). In previous experiments, the lethal and innocuous doses of each bacterium were determined (Supplementary Figure S3). Based on these results, the highest safe doses were selected: 10^4^ CFU/larva for *M. abscessus* R and S, and 10^5^ CFU/larva for *B. thuringiensis* and *E. coli*. In the case of *P. aeruginosa*, even the lowest tested dose (10 CFU/larvae) was lethal. The virulence of the *P. aeruginosa* PAO1 strain was much higher than that of PAET1; therefore, the PAET1 strain dose was adjusted to 10^4^ CFU/larva, similar to the 10 CFU/larva of PAO1. To confirm *P. aeruginosa* infection, bioluminescence images of luciferase in infected larvae were obtained using Image Quant LAS 4000 (GE Healthcare, Chicago, USA) (Supplementary Figure S4). Thirty larvae were infected under each condition, *E. coli*, *B. thuringiensis* and *M. abscessus* R/S, and were then injected with the *P. aeruginosa* PAO1 or PAET1 strains. Additionally, larvae infected with only one *P. aeruginosa* strain were used as controls for comparison purposes. Infected larvae were maintained at 37°C for 14 h to allow for the development of infection. Five larvae were used to extract RNA, and 30 were monitored for survival for 24 h.

To analyze the phagocytosis of *M. abscessus* R and S by *G. mellonella* hemocytes and the concentration of mycobacteria in the hemolymph, larvae were infected with 10^4^ CFU/larvae of each variant. For hemolymph collection, larvae were placed on ice for 10 min. Once anesthetized, the anal proleg was cut with a surgical blade and the hemolymph was collected in a 1.5 ml Eppendorf tube. Hemolymph from 10 larvae were pooled, appropriately diluted, and cultured on TSA plates for mycobacterial CFU counts. The hemolymph from the other 10 larvae was centrifuged and washed three times with PBS (5 min at 500 g at 4°C). Precipitated hemocytes were resuspended in PBS and stained with DAPI (blue) (4′,6-diamidino-2-phenylindole) to observe nuclei, and with FM 4–64 (red) (*N*-3-triethylammoniumpropyl-4-6-4-diethylamino phenyl hexatrienyl pyridinium dibromide) to observe dying hemocyte vesicles and plasma membrane under a confocal microscope (Zeiss LSM 800 confocal laser scanning microscope (CSLM)).

### *G. mellonella* RNA extraction and immune-relevant gene expression analysis

At 14 h post-infection, five larvae were placed on ice for 10 min. The larvae were homogenized using a rotor-stator homogenizer (IKA, Staufen, Germany), and 30 mg of the homogenate was used for RNA extraction using a GeneJET RNA Purification Kit (Thermo Fisher Scientific). The RNA obtained was treated with 10× TURBO DNase (Life Technologies, Carlsbad, CA, USA) for 1 h to eliminate possible DNA contamination. The absence of DNA was verified by PCR amplification of the 18S rRNA housekeeping gene using the genomic DNA of *G. mellonella* as a positive control. The RNA was quantified using an M200 PRO microplate reader (Tecan). RNA to cDNA reverse-transcription was performed using Maxima Reverse Transcriptase (Thermo Fisher Scientific) with Oligo (dT)18 primers (Thermo Fisher Scientific), according to the manufacturer’s instructions. The cDNA obtained was stored at −20°C until use. Quantitative real-time PCR (qRT-PCR) was performed using PowerUp™ SYBR™ Green Master Mix (Applied Biosystems, Foster City, CA, USA) on a StepOnePlus™ Real-Time PCR System (Applied Biosystems) according to the manufacturer’s protocol. All qRT-PCRs used specific primers for *G. mellonella* immune-relevant genes listed in Supplementary Table S2.

### Bronchial epithelial cell line culture and infection

Two human bronchial epithelial cell lines were used: CFBE41o-, isolated from a patient with CF homozygous for the ΔF508 CFTR mutation, and 16HBE14o-, isolated from a cardiopulmonary patient overexpressing CFTR [38]. Cells were maintained in Dulbecco’s modified Eagle’s medium: Nutrient Mixture F12 (DMEM/F12; Thermo Fisher Scientific) with 10% (v/v) decomplemented fetal bovine serum (dFBS) (Gibco, Paisley, UK) and 1% (v/v) penicillin‒streptomycin (Thermo Fisher Scientific). The cells were maintained in a humidified incubator at 37°C with 5% (v/v) CO_2_ (ICOmed, Memmert). For infection experiments, cells (5×10^4^ cells/well) were incubated in 96-well plates (Corning) in antibiotic- and serum-free medium for 3 h to allow cell adhesion. Subsequently, the cells were infected at a multiplicity of infection (MOI) of 50:1 for *P. aeruginosa* PAO1 and PAET1 and 10:1 for *M. abscessus* R/S and *B. thuringiensis*. Infections with each bacterium, as well as coinfections combining both strains of *P. aeruginosa* and *M. abscessus* were carried out and plates were leaved in the incubator 37°C with 5% CO_2_. Three hours later, the wells were washed 3 times with PBS to remove extracellular bacteria, refilled with fresh antibiotic-free media, and the plates were incubated for 24 h. The supernatant was then removed and stored at −80°C for cytokine measurements. The effects of *P. aeruginosa*, *M. abscessus* and their combination on cell viability were analyzed using the Presto Blue assay (Thermo Fisher Scientific) following the manufacturer’s instructions, using uninfected cells as a control. Cell infections were performed in triplicates in three independent experiments.

### IL-6 and IL-8 detection by enzyme immunoassays (ELISAs) in cell supernatants

IL-6 (Cat. No. 555220) and IL-8 (Cat. No. 555244) production in cell culture supernatants was measured using ELISA kits according to the manufacturer’s instructions (Becton Biosciences, BD, San Diego, USA). The assays BD OptEIA™ Human IL-6 and IL-8 detect concentrations ranging from 4.7–300 pg/mL and 3.1–200 pg/mL, respectively. The supernatant samples were appropriately diluted to ensure their detection within these ranges. Briefly, wells were coated with IL-6 or IL-8 capture antibody diluted in coating buffer (0.1 M sodium carbonate, pH 9.5) ON at 4°C. The plates were then washed and blocked with an assay diluent (PBS with 10% FBS) for 1 h at room temperature (RT). Plates were prepared with standards diluted in the assay diluent, and the samples were incubated for 2 h at RT. After washing, streptavidin-horseradish peroxidase conjugate mixed with biotinylated detection antibody was added to each well for 1 h. Finally, tetramethylbenzidine (TMB) substrate solution and hydrogen peroxide (BD, San Diego, USA) were added to the plates for 30 min in the dark. Absorbance (OD_630 nm_) was measured in an Infinite 200 PRO microplate reader (Tecan) and analyzed using the Magellan software (Tecan).

### Data statistical analysis

All data were analyzed using appropriate statistical methods based on the experimental design of each approach. Results were expressed as mean ± standard deviation (SD) of at least three independent experiments. For all statistical tests, a *p*-value < 0.05 was considered statistically significant. Data processing and analyses were conducted using GraphPad Prism software.

**Figure 1.**
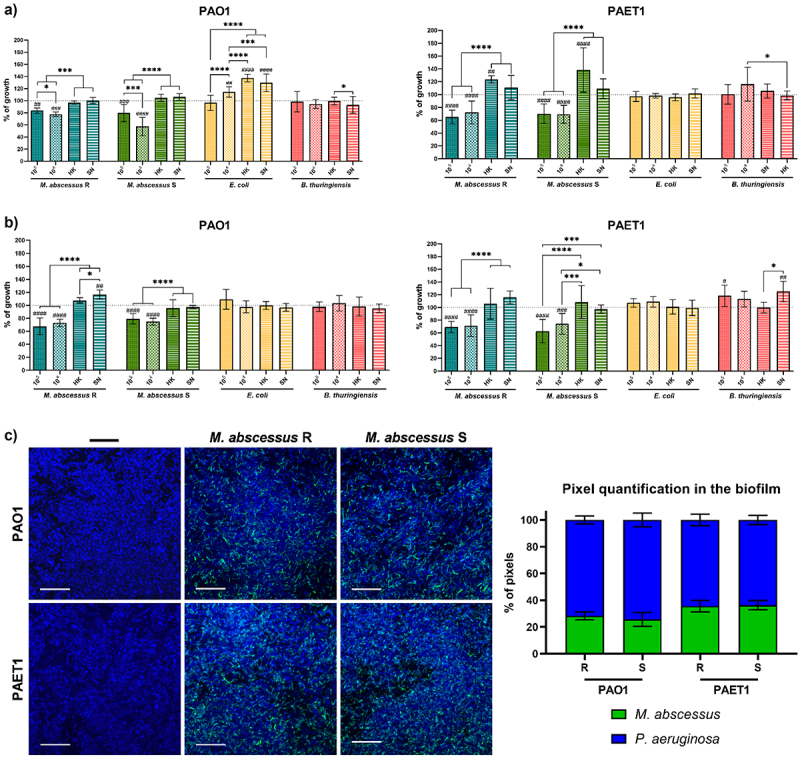
Coculture of *P. aeruginosa* PAO1 and PAET1 biofilms with different bacteria. (a) Growth of well-developed mature *P. aeruginosa* biofilm when different bacterial cultures (live and heat-killed) or bacterial culture supernatants were added. (b) Growth of *P. aeruginosa* in coculture biofilms. In both a) and b) the values correspond to the luciferase expression (*lux*) of the *P. aeruginosa* strains generated in each condition, normalized based on the average expression reported in control wells (marked with a dashed line as 100% *P. aeruginosa* growth). The effects of *M. abscessus* R (rough) and S (smooth) morphotypes, *E. coli* MG1655 and *B. thuringiensis* (10^4^ /10^5^ CFUs/well) on both *P. aeruginosa* strains were tested as well as the supernatant (SN) and heat-killed (HK) form of these bacterial cultures. Data are presented as the mean values ± SDs of at least three independent experiments including six replicates in each experimental condition; significant differences were determined using one-way ANOVA and Tukey’s multiple comparisons tests; ****, *p* < 0.0001; ***, *p* < 0.001; *, *p* < 0.05. Comparison of cocultures with individual PAO1/PAET1 biofilms: ^*####*^, *p* < 0.0001; ^*###*^, *p* < 0.001; ^##^, *p* < 0.01; *^#^, p* < 0.05. (c) Representative confocal microscopy images of coculture biofilms and pixel color quantification of the images. *P. aeruginosa* PAO1 and PAET1 biofilms were grown for 72 h, and *M. abscessus* R and S were inoculated for an additional 24 h. Z-stack compositions of 96 h-old *P. aeruginosa* PAO1 and PAET1 biofilms in combination with *M. abscessus* R and S morphotypes. DAPI stained all cells blue, and green fluorescent protein (GFP) of plasmid pETS218 allowed mycobacterial cells to be detected in green. Scale bars correspond to 20 µm. Pixel quantifications are represented as the mean values ± SDs from three representative images of each condition.

**Figure 2.**
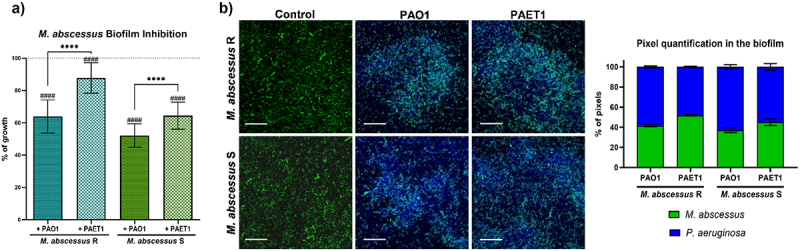
Effect of the *P. aeruginosa* PAO1 and PAET1 strains on *M. abscessus* R (rough) and S (smooth) biofilm development. (a) Graph represents the inhibition of *M. abscessus* biofilms when cocultured for 72 h with *P. aeruginosa* by measuring green fluorescent protein (GFP) expression. The control corresponds to 100% of single *M. abscessus* biofilm growth by analyzing GFP expression (dotted line). Data are presented as the mean ± SD of three independent experiments including six replicates in each experimental condition, and significant differences were reported using the Mann‒Whitney *t* test, ****, p < 0.0001. Comparison of cocultures with individual *M. abscessus* biofilms: ^*####*^, *p* < 0.0001. (b) Confocal microscopy representative coculture biofilm images and pixel color quantification. Z-stack compositions of *M. abscessus* variant 120 h biofilms in combination with *P. aeruginosa* strains. DAPI (4′,6-diamidino-2-phenylindole) stained all cells blue, and GFP of plasmid pETS218 allowed mycobacterial cells to be detected in green. Scale bars correspond to 20 µm. Pixel quantifications are represented as the mean values ± SDs from three representative images of each condition.

**Figure 3.**
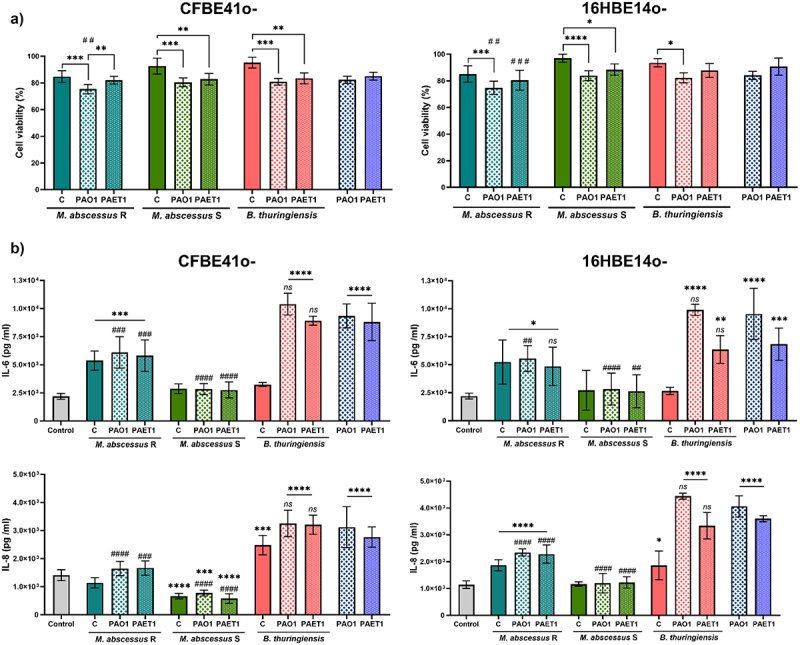
Bronchial epithelial cell viability and proinflammatory cytokine production after *P. aeruginosa* PAO1/PAET1, *M. abscessus* R (rough)/S (smooth) and *B. thuringiensis* infections. (a) Viability of CFBE41o- and 16HBE14o- cell lines after bacterial infections. Presto blue cell viability assay results in which data are expressed with respect to uninfected wells (considered as 100% cell viability). Data are represented as the mean ± SD from three independent experiments. Significance was analyzed using one-way ANOVA and Tukey’s multiple comparisons test. *, *p* < 0.05; **, p < 0.01; ***, p < 0.001; ****, p < 0.0001. Comparison of cocultures with individual PAO1/PAET1 infections: ^##^, *p* < 0.01; ^*###*^, *p* < 0.001. (b) Interleukins 6 and 8 (IL-6 and IL-8) production detected in culture supernatants from bronchial epithelial cell infections. Statistical differences were determined using one-way ANOVA and Dunnett’s test, which compares each column with the control (uninfected cells). Data are represented as the mean ± SD from three independent experiments; *, *p* < 0.05; **, p < 0.01; ***, p < 0.001; ****, p < 0.0001. Comparison of results obtained in PAO1/PAET1 individually infected cells with coinfections were determined using one-way ANOVA, Dunnett’s test comparisons test: *ns*, non-significant; ^##^, *p* < 0.01; ^*###*^, *p* < 0.001; ^*####*^, *p* < 0.0001.

**Figure 4.**
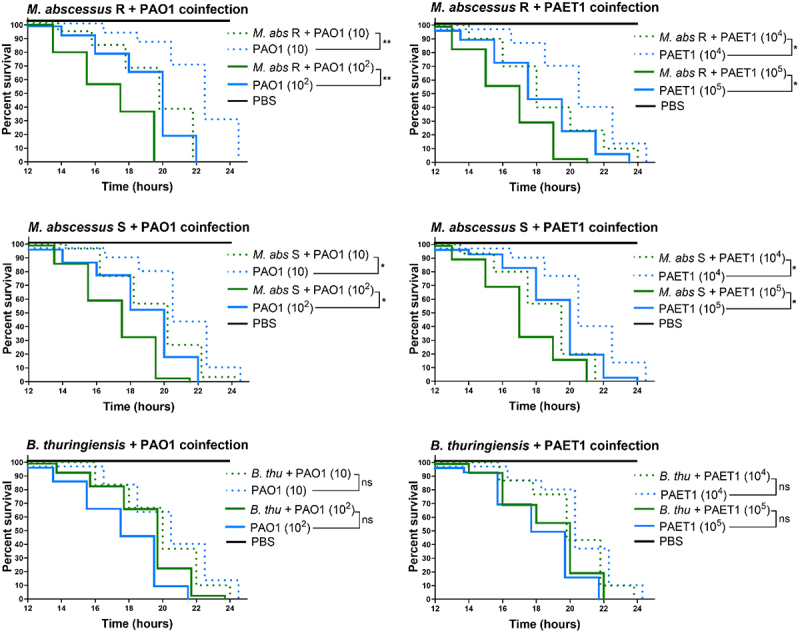
*B. thuringiensis* and *M. abscessus* coinfecting with *P. aeruginosa* in *G. mellonella* Kaplan – Meier survival curves. Thirty larvae (*n* = 30) of each condition were monitored from 12 h post-infection every 2 hours to verify their survival rates when *M. abscessus* rough (R) and smooth (S) morphotypes (10^4^ CFU/larva) or *B. thuringiensis* (10^5^ CFU/larva) was coinfected with *P. aeruginosa* PAO1 and PAET1 at different concentrations. Phosphate buffered saline (PBS) was used as safe survival control. Data were analyzed by comparing the curves of individual *P. aeruginosa* infections with coinfection of *M. abscessus* plus *P. aeruginosa* at the same concentration using the Mantel‒Cox survival test; *, p < 0.05; **, p < 0.005.

**Figure 5.**
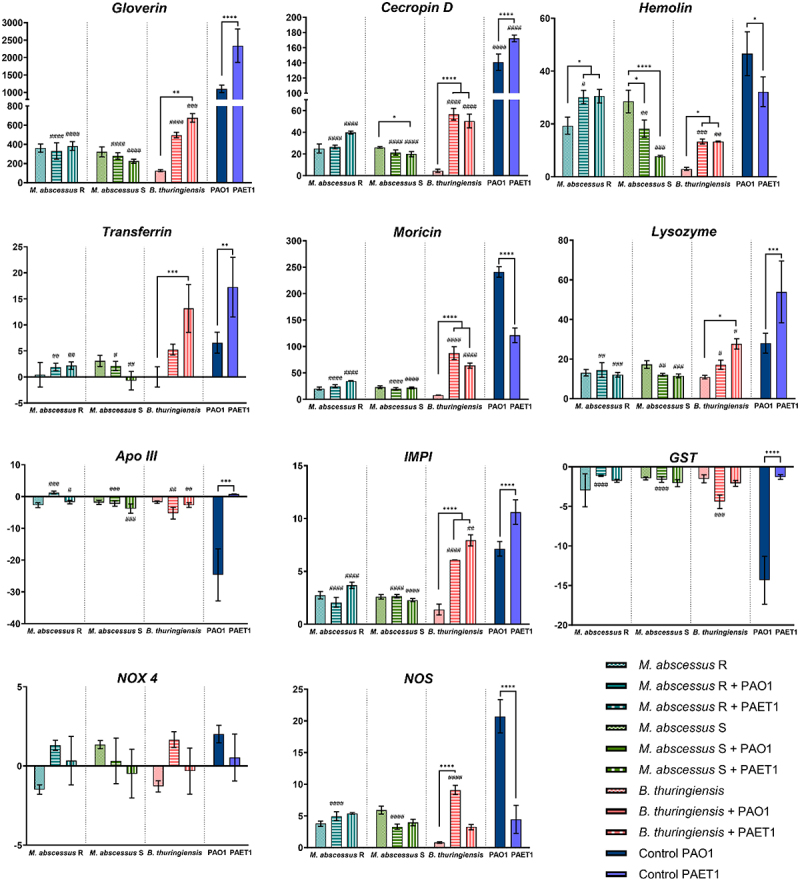
Expression of *G. mellonella* immune-relevant genes under different infection conditions. The fold changes indicate the number of times above or below the gene expression compared with the control (larvae infected with PBS) obtained by RT‒PCR analysis. Data are presented as the mean ± SD of the fold changes for each condition. Significant differences of individual *B. thuringiensis* and *M. abscessus* (R/S) infection *vs* coinfections were established using one-way ANOVA Tukey’s multiple comparisons test. *, *p* < 0.05; **, p < 0.01; **, p < 0.001; ****, p < 0.0001. Significant differences of individual *P. aeruginosa* (PAO1/PAET1) *vs* coinfections were established using one-way ANOVA Tukey’s multiple comparisons test. #, *p* < 0.05;* ##, p* < 0.01;* ###, p* < 0.001;* ####, p* < 0.0001. *Apo III*: Apolipophorin III, *IMPI*: insect metalloproteinase inhibitor, *GST*: glutathione S-transferase, *NOX 4*: NADPH oxidase, *NOS*: nitric oxide synthase.

In the *P. aeruginosa* PAO1 and PAET1 coculture biofilms (Figure 1), the luciferase expression (*lux* intensity) was normalized to control individual biofilms (set as 100%). Significant differences in biofilm growth under different coculture conditions and were determined using one-way ANOVA followed by Tukey’s multiple comparisons test. In the biofilm inhibition of *M. abscessus* (Figure 2) GFP intensity measurements of *M. abscessus* R and S biofilms were normalized to single-strain *M. abscessus* biofilms (set as 100%). Significant differences were assessed using the Mann-Whitney *t*-test, with annotations for cocultures versus single-strain *M. abscessus* biofilms. For both Figures 1 and 2 confocal microscopy data were analyzed similarly, with mean ± SD calculated from representative images. The bronchial epithelial cell viability and cytokine production (Figure 3) data from Presto Blue assays were normalized to uninfected control wells (set as 100%). Cytokine production (IL-6 and IL-8) was analyzed using one-way ANOVA with Dunnett’s test to compare each infection condition with uninfected controls, and differences between individual and coinfection conditions. *G. mellonella* survival curves (Figure 4) were analyzed using the Mantel-Cox log-rank test, and comparisons were made between single and coinfection conditions, with significant differences reported. Finally, *G. mellonella* fold changes in immune gene expression (Figure 5) were calculated relative to PBS-infected controls. Differences between single infections and coinfections were evaluated using one-way ANOVA with Tukey’s multiple comparisons test.

## Results

### *M. abscessus* reduces the progression of *P. aeruginosa* biofilms in vitro

*In vitro* biofilm development assays showed that the addition of *M. abscessus* R or S to mature *P. aeruginosa* biofilms limited their growth capacity in terms of lower luciferase expression (Figure 1(a)). This inhibitory effect was observed for both *P. aeruginosa* PAO1 and PAET1 biofilms. Specifically, the presence of *M. abscessus* (R and S) resulted in a growth reduction of approximately 20% for PAO1 and 30% for PAET1 biofilms (Figure 1(a)) compared with non-treated *P. aeruginosa* biofilms luciferase measurements (represented as 100% growth). Nevertheless, the highest rate of growth inhibition was reported for *M. abscessus* S at 10^4^ CFU/well, with approximately 40% PAO1 biofilm *lux* reduction. The addition of heat-killed *M. abscessus* or bacterial culture supernatants had no inhibitory effect on the growth of *P. aeruginosa* mature biofilms (Figure 1(a)). Similarly, the use of *E. coli* (gram-negative) or *B. thuringiensis* (gram-positive) did not have a growth-limiting effect on *P. aeruginosa* biofilms *lux* expression, and even in the case of heat-killed *E. coli* and supernatant from *E. coli* cultures, it induced *P. aeruginosa* PAO1 biofilm growth producing higher *lux* intensities (Figure 1(a)).

When *P. aeruginosa* strains were cocultured simultaneously with all bacteria tested, the results were comparable with the previous assay results, but with slight inhibition percentage changes in some cases (Figure 1(b)). *P. aeruginosa* PAO1 cocultured with the *M. abscessus* R morphotype showed a 30% reduction in PAO1 biofilm *lux* expression compared to the control, while with the S morphotype, the reduction in PAO1 biofilm *lux* expression was only approximately 20%. In *P. aeruginosa* PAET1 cocultures, the results using *M. abscessus* R or S were quite similar, with an approximately 30% decrease luciferase activity in the PAET1 biofilm compared to the control (growth of *P. aeruginosa* alone) (Figure 1(b)). As shown in Figure 1(a), the addition of *E. coli* and *B. thuringiensis* and the use of heat-killed cultures or supernatants did not have an inhibitory effect on the *P aeruginosa* PAO1 and PAET1 biofilms luciferase intensities (Figure 1(b)). In addition, it was observed that PAET1 biofilm progression was more inhibited in coculture with *M. abscessus* (R and S) than that of PAO1 in all the biofilm assays tested.

After observation of a clear *P. aeruginosa* biofilm reduction in luciferase expression with the addition of *M. abscessus*, either added to a mature biofilm or when simultaneously grown in a dual-species biofilm, the structure and composition of coculture biofilms were analyzed by confocal microscopy. The images showed that after 24 h of coculture, *M. abscessus* was established in a *P. aeruginosa* mature biofilm, demonstrating the capacity to settle and simultaneously grow under these conditions (Figure 1(c)). The presence of *P. aeruginosa* cells (in blue) was clearly higher, but *M. abscessus* bacilli (in green) were clearly observed in both *P. aeruginosa* PAO1- and PAET1-formed biofilms and quantitatively represented between 25% and 35% of the sample, as shown in the pixel color quantification graph (Figure 1(c)). This result demonstrates that both species can survive and coexist in dual-species biofilms. Apparently, the presence of both morphotypes R and S of *M. abscessus* was higher in the *P. aeruginosa* PAET1 biofilm than in PAO1, consistent with the results obtained in Figure 1(a), where growth inhibition was higher for the PAET1 strain under the same experimental conditions as for the PAO1 strain. The structure of the biofilm was observed through orthogonal projections that *P. aeruginosa* and *M. abscessus* were heterogeneously distributed along the biofilm without predominantly occupying any specific area (Supplementary Figure S2).

In view of the results obtained, a possible direct inhibitory effect between *P. aeruginosa* PAO1/PAET1 growth and some molecules released by *M. abscessus* R and S, *E. coli* or *B. thuringiensis* was tested. For this purpose, all bacteria were cultivated on agar plates in closed streaks, where no inhibitory effects were observed on the growth of any bacteria in the presence of the others (Supplementary Figure S5(a)). Kinetic curves of *P. aeruginosa* PAO1 and PAET1 planktonic growth were also generated by cocultivation with culture supernatants of the other bacteria, and similar to the solid agar cultures, no significant inhibitory effect on the growth of *Pseudomonas* was reported (Supplementary Figure S5(b,c)).

### *P. aeruginosa* reduces the development of *M. abscessus* biofilms

Although infection by *M. abscessus* in CF patients generally occurs in patients previously infected with *P. aeruginosa*, we aimed to study the effect of *P. aeruginosa* on well-developed *M. abscessus*. Over a mature *M. abscessus* biofilm (120 h old), the addition of *P. aeruginosa* was directly related to a clear reduction in the development of the *Mycobacterium* biofilm in terms of lower GPF intensities (both for R and S biofilms) (Figure 2(a)), similar to the opposite case (Figure 1(a)). Biofilm inhibition was notably higher in the *M. abscessus* S morphotype than in the R morphotype under all conditions tested. Additionally, *P. aeruginosa* PAO1 induced a higher growth inhibitory effect in both *M. abscessus* biofilm morphotypes than *P. aeruginosa* PAET1. The results obtained by GFP quantification were corroborated by confocal microscopy (Figure 2(b)). The images show that after treatment of an *M. abscessus* biofilm with *P. aeruginosa*, both bacteria form a combined biofilm in which *P. aeruginosa* expands rapidly due to its competitive advantage in terms of growth rate. The detection of mycobacteria by GFP in coculture conditions was reduced compared with that of the control biofilms (without *P. aeruginosa*), and a lower presence of *M. abscessus* was also detected in the S morphotype compared to the R morphotype in combination with both *P. aeruginosa* PAO1 and PAET1 (Figure 2(b)).

### Coinfection of *M. abscessus* and *P. aeruginosa* in bronchial epithelial cells reduces the viability and immune response triggered with respect to single *P. aeruginosa* infection

To determine the effect of bacterial coinfection in the CF context, CFBE41o- (homozygous for the ΔF508 CFTR mutation) and 16HBE14o- (usually differentiated bronchial epithelial cells) bronchial epithelial cells were infected with *P. aeruginosa*, *M. abscessus, B. thuringiensis* or a combination of bacteria. The results showed a reduction in viability of approximately 10–30% in infected cells compared to uninfected cells in all cases (Figure 3(a)). Remarkably, while single infection with *M. abscessus* variants or *B. thuringiensis* reduced viability by up to 5–15% in both cell lines, when cells were coinfected with either of the two *P. aeruginosa* strains, viability was significantly diminished with respect to single infection (Figure 3(a)). In all cases, the viability levels of coinfected cells were similar to those obtained with a single infection of *P. aeruginosa* strains, with the exception of the *P. aeruginosa* strains PAO1 and PAET1 and *M*. *abscessus* R-coinfected cells, in which lower viability was obtained (Figure 3(a)). No differences were observed between the cell lines, indicating that the inhibitory effect of the bacteria was not dependent on epithelial characteristics.

Differences were observed for each culture condition when IL-6 and IL-8 production levels were analyzed. When cytokine production was analyzed in cell culture supernatants, *P. aeruginosa*-infected cell lines showed the highest levels, indicating an elevated inflammatory response (Figure 3(b)). Single infections with *M. abscessus* R, S, and *B. thuringiensis* showed different stimulatory capabilities, whereas *M. abscessus* S did not induce the production of cytokines, *B. thuringiensis* triggered only IL-8 production in both cell lines, *M. abscessus* R triggered IL-6 production in both cell lines, and IL-8 only in the control cell line (16HBE14o-) (Figure 3(b)).

The most relevant result was observed in cells coinfected with both bacteria. *M. abscessus* coinfection was able to inhibit the higher production that was reported in *P. aeruginosa* single-infected cells, and the release of IL-6 and IL-8 was reduced to levels similar to those triggered by *M. abscessus* single infection (Figure 3(b)). *M. abscessus* S and *P. aeruginosa* (both strains) coinfection reduced the production of cytokines to levels similar to those in the control uninfected cells. Strikingly, this effect observed with *M. abscessus* was not observed when *B. thuringiensis* was coinfected with *P. aeruginosa*, triggering cytokine levels similar to those produced by *P. aeruginosa* single infection (Figure 3(b)). No differences were observed between the cell lines.

### *M. abscessus* and *P. aeruginosa* coinfection accelerates *G. mellonella* larval death

The *G. mellonella* larvae *in vivo* model present an immune system broadly similar to mammalian innate immunity, which is useful for the characterization of microbial infections and host-pathogen interactions. Infection with both *P. aeruginosa* PAO1 and PAET1 strains is lethal to *G. mellonella* larvae. However, *M. abscessus* infections require prolonged time (up to 144 h) and high concentrations (10^6^ CFU/larva) to become lethal (see Materials and Methods and Supplementary Figure S3). Coinfection with *M. abscessus* R and S, but not with *B. thuringiensis* accelerated the survival decline in the larvae in both strains and all concentrations tested compared to the infections of *P. aeruginosa* strains alone (Figure 4). This overtaking lethality was clearly observed, being significant for all *M. abscessus* and *P. aeruginosa* coinfection combinations tested, especially in the case of PAO1 plus *M. abscessus* R coinfection. We corroborated that the causal agent of larval death was the presence of *P. aeruginosa* PAO1 or PAET1 in the larvae by detecting luminescence in their interior through *lux* detection in a luminescence analysis (Supplementary Figure S4). As Figure 4, *B. thuringiensis* coinfection did not affect larval survival rates.

### *M. abscessus* and *P. aeruginosa* coinfection restricts the immune response of *G. mellonella* larvae

*G. mellonella* larvae produce several antimicrobial peptides (AMPs) and enzymes in response to infection. The results of immune-relevant *G. mellonella* gene expression showed a significant decrease in the transcriptional induction of several genes tested, comparing *P. aeruginosa* individual infections with different coinfection conditions (Figure 5). For gloverin, cecropin D, transferrin, moricin, lysozyme, insect metalloproteinase inhibitor (IMPI) and nitric oxide synthase (NOS) gene expression, a comparable trend was observed between all infections assayed. Briefly, high induction of gene expression in PAO1- and PAET1-infected larvae was observed, with lower induction of gene expression in larvae coinfected with *B. thuringiensis* and *P. aeruginosa* strains and notably lower gene expression in the case of larvae infected with both *M. abscessus* R and S alone and coinfected with *P. aeruginosa* (Figure 5). These data indicated that the presence of *M. abscessus* generated a decrease in gene expression compared with the infection of single *P. aeruginosa* strains, leaving the induction of larvae coinfected by both bacteria at the levels established by the mycobacteria alone.

Apolipophorin III (Apo III) and glutathione S-transferase (GST) showed a similar trend, with no significant differences detected compared to the control (PBS-infected larvae) in all conditions except for the *P. aeruginosa* PAO1-infected larvae, which showed evident repression of both genes. NADPH oxidase (NOX4) gene expression was the only gene that did not show significant differences in any of the infections tested. In the case of hemolin, increased gene expression was observed in *M. abscessus*-infected larvae, which was much higher than in the other studied genes and *B. thuringiensis* infection conditions. This result contradicts other immuno-relevant gene trends. Hemolin acts as an opsonin and mediates the interaction between microorganisms and hemocytes, stimulating phagocytosis. In this study, we also showed that *M. abscessus* R and S could be phagocytosed by *G. mellonella* hemocytes (Supplementary Figure S6(a)) and progressively eliminated from the larva (Supplementary Figure S6(b)), which would explain this contradictory result compared with the rest of the genes.

## Discussion

Infections of the respiratory tract epithelium are the leading cause of morbidity and mortality in patients with CF, as well as other respiratory airway diseases commonly associated with infections such as bronchiectasis, chronic obstructive pulmonary disease, asthma, etc. *P. aeruginosa* is the most relevant pathogen owing to its robust virulence systems and biofilm-forming capacity, which triggers increased tolerance to antibiotics and resistance to phagocytosis [10,39]. Although *P. aeruginosa* is the most common and prevalent pathogen in patients with CF, other microorganisms, such as *S. aureus*, *Haemophilus influenzae*, *Stenotrophomonas maltophilia*, NTM, and *Burkholderia* species, are presently forming a polymicrobial environment. Within the NTM group, *M. abscessus* is the most relevant pathogen in CF patients because its multidrug-resistant and biofilm-forming capabilities [23,40] widely increase its antibiotic resistance and prevalence. In this study, the relationship between *P. aeruginosa* PAO1 (laboratory strain)/PAET1 (clinical isolate) and *M. abscessus* R/S morphotypes was studied for the first time in different contexts of critical interest: biofilm development, bronchial epithelial cell *in vitro* culture, and *G. mellonella* larvae *in vivo* infection.

Regarding biofilm development, the results indicated a decrease in the progression of *P. aeruginosa* biofilms when live *M. abscessus* R and S were introduced, independent of whether the biofilm was already well established (mature biofilm) or in the coculture biofilm growth. Inhibition of *Pseudomonas* biofilm development by cocultivation with other pathogens commonly found in the lungs of CF patients, such as *Aspergillus*, or potential probiotics, such as lactobacilli, has already been reported [41,42]. Nevertheless, no inhibitory effect has been previously reported for mycobacteria. This inhibition could be related to the ability of *M. abscessus* to inactivate quorum sensing quinolone signals from *P. aeruginosa* [43]. In addition, the results showed that both species coexist in dual-species biofilms, with *P. aeruginosa* being the dominant species, most likely related to its competitive advantage in terms of growth, since *M. abscessus* has a division time of 4–5 h, while *P. aeruginosa* has a division time of only 25–35 min [44,45]. Conversely, biofilms of *M. abscessus* were also inhibited by the presence of *P. aeruginosa*, which outcompetes mycobacteria, as previously reported in biofilm coculture experiments [46]. The mechanism responsible for this inhibition remains unknown; however, well-known mechanisms of interbacterial competition (secretion of antagonist factors, quorum sensing quinolone signals, motility, iron sequestration systems, etc.) are not involved, suggesting a novel antibacterial strategy [47]. Distinctly, the growth of S-morphotype biofilms, despite forming biofilms more easily by the abundance of GPLs in its cell wall [48], was inhibited more by the presence of *P. aeruginosa* than R morphotype biofilms, which could be related to the aggregative phenotype that confers higher resistance to antibiotics as well as structural rigidity [49]. These results indicated a decrease in biofilm progression in both species when cocultured. It can be hypothesized that this effect could not be due to direct inhibition but rather a consequence of the combined growth of both species sharing the same space and competing for the same culture medium. This hypothesis is partly supported by the lack of direct inhibition observed in agar plate cultures using *M. abscessus* R and S cultured close to *P. aeruginosa* PAO1 and PAET1, or in planktonic growth kinetics curves of *P. aeruginosa* PAO1 and PAET1 in combination with culture supernatants of the remaining bacteria, in which no significant effect on the growth of *P. aeruginosa* was highlighted (see Supplementary Figure S5). It has been demonstrated that, in dual-species biofilms, antibiotic treatment to eliminate *P. aeruginosa* is a competitive advantage for *M. abscessus* development [20]. In any case, *M. abscessus* establishes itself effectively, and despite its difference in growth rate, prevails in dual-species biofilms, demonstrating its resistance and survival capabilities [50].

Regarding the effect of coinfection on bronchial epithelial cell viability *in vitro*, it has been shown that coinfection results in higher mortality than individual *M. abscessus* and *B. thuringiensis* infections, as expected. However, as previously reported [38], the viability of the cell lines tested, CFBE41o- and 16HBE14o-, was not excessively compromised, being approximately 80% of the control under all tested conditions. *M. abscessus* and *P. aeruginosa* coinfections correlate with an elevated pulmonary function decline and worse disease severity [51,52]; therefore, lower viability of epithelial cells could be involved in these complications. The inflammatory response triggered by PAO1 and PAET1 single infections in epithelial cells clearly exacerbated the production of the proinflammatory cytokines IL-6 and IL-8 (Figure 3). This response is due to activation of the Toll-like Receptor (TLR). *P. aeruginosa* lipopolysaccharide is recognized by TLR2 [53,54], leading to high proinflammatory cytokine production [55]. In addition, flagellin-mediated signaling by TLR5 is a clue in *P. aeruginosa* infection, and this is of particular interest because in the case of airway epithelial cells from CF patients, TLR5 mRNA expression is increased [56].

*P. aeruginosa* produces virulence factors, such as proteases and elastases, which can degrade host immune signaling molecules like cytokines and components of the complement system [57], potentially protecting also *M. abscessus* in coinfections. Furthermore, *P. aeruginosa* can produce immunomodulatory virulence factors such as pyocyanin and rhamnolipids, which suppress the initial proinflammatory Th1 cytokine production (as IFN-γ) and upregulate Th2 cytokine response, favouring chronic infections as demonstrated in CF patients [58]. In this sense, Th1 responses are crucial for effective mycobacterial infection control, and *M. abscessus* may benefit from this Th2 polarization [59]. This immune response against *P. aeruginosa* is harmful to patients, as excessive inflammation is responsible for most of the morbidity and mortality associated with CF [60–62]. Surprisingly, coinfection of *M. abscessus* and *P. aeruginosa* with epithelial cells showed significant inhibition of the proinflammatory response promoted by the mycobacteria, since single *M. abscessus*-infected cells triggered similar cytokine levels as the coinfected cells. The bronchial epithelial cell hyporesponsiveness triggered by *M. abscessus* S could be due to the TLR2 blocking effect of the GPLs present in the cell wall of this morphotype [63], since IL-6 and IL-8 release in *M. abscessus* infection is TLR2 dependent [64,65]. The presence of GPLs in the cell wall masks phosphatidyl-myo-inositol mannosides (PIMs), thereby blocking interactions with TLR2 [63]. This inhibition of pro-inflammatory signalling facilitates immune evasion and colonization. This aligns with our findings, which indicate no significant differences in IL-6 and IL-8 levels between *M. abscessus* S infected and uninfected controls in both CFBE41o- and 16HBE14o- bronchial epithelial cells. Notably, IL-8 levels in CFBE41o- cells were significantly lower in *M. abscessus* S infected samples compared to uninfected cells. In contrast, the rough (R) morphotypes of *M. abscessus* (lacking GPLs) stimulate TLR2 and upregulate IL-6 and IL-8 expression [63], correlating with a significant increase in the production of these cytokines compared to uninfected controls in our study. However, the release of IL-6 and IL-8 in individual *M. abscessus* R infections matches those observed with *P. aeruginosa* coinfections, indicating a potential unknown mechanism of non-GPL related proinflammatory suppression.

Microorganisms that occupy the airways together with *P. aeruginosa* also induce a proinflammatory response, as in the case of *B. cenocepacia, H. influenzae, S. aureus* and *Candida spp*. among others [66,67]. A comparable *M. abscessus-*triggered reduced inflammatory response has only been demonstrated in *P. aeruginosa* coinfections with *Prevotella spp*., an anaerobic gram-negative bacterium belonging to the respiratory tract microbiota [68]. TLR signaling by *Prevotella histicola* activates alternative NF-κB signaling in CF bronchial epithelial cells compared with *P. aeruginosa*. Data from Bertelsen et al. [69] suggested that *Prevotella spp*. could use other non-flagellin structures for TLR5 engagement. TLR5 agonists could be present in *M. abscessus*. Indeed, there is another relevant example in bacterial pathogens: masking canonical TLR5 agonists with structures of advantageous characteristics provides more flexibility to *Helicobacter pylori* in evading host immune responses [70]. Finally, it has recently been demonstrated that TLR5 potentiates LPS-mediated TLR4-MyD88 signaling through physical interactions [71]. Thus, antagonists of TLR5 could also influence the reduction in TLR4-induced inflammatory cytokines. In fact, some glycolipids [72] and proteins from *M. tuberculosis*, such as ESX proteins [73], are TLR2 antagonists that inhibit TLR2-mediated NF-kB activation. It is plausible that the low immune response observed in *M. abscessus* – *P. aeruginosa* coinfection follows some of these previous strategies. Further research is warranted to elucidate the underlying mechanism and identify the molecules responsible for this inflammatory signaling block.

Overall, cocultures of *M. abscessus* and *P. aeruginosa* showed less inflammatory activation and the highest loss of viability among infected cells, while cocultures of *B. thuringiensis* and *P. aeruginosa* showed a high inflammatory response, leading to a high loss of viability. These results indicated that an excessive proinflammatory response and significant immune inhibition could be equally harmful to infected lung epithelial cells. In the context of CF, a beneficial immune response consists of the capacity to activate an inflammatory response to manage infection but to avoid an exuberant response that can be harmful [74], as supported by our *in vitro* results. In this regard, immune suppression caused by coinfection with *M. abscessus* and *P. aeruginosa* could lead to promote persistent infections due to the limited host ability to clear the bacteria, collectively accelerating lung function decline and increasing therapy resistance in CF patients. Besides in the present work laboratory research strains have been used as a reference, it would be positive to introduce a broader range of strains, especially clinical isolates, to be able to contrast the results to a greater extent and enhance the translational relevance. The results of the *in vitro* cultures were corroborated by infections in the *G. mellonella* larva model, in which increased lethality was observed in coinfection conditions with respect to the single *P. aeruginosa* infection. *G. mellonella* is highly susceptible to *P. aeruginosa* infections because 100% larval lethality is reached in less than 24 h with a few inoculated bacteria (10–30 CFU) [75,76], although there are important variations in pathogenicity and lethality between strains commonly used in research (PAO1 in this study) and clinical isolates (PAET1 in this study) [38,77]. However, *M. abscessus*, despite being pathogenic to larvae, requires much higher doses and prolonged infection times than *P. aeruginosa* to be equally lethal ([78,79] and Supplementary Figure S3). In addition, in agreement with our *in vitro* results, the immune response of *G. mellonella* larvae was clearly inhibited under coinfection conditions (Figure 5). The gene expression of most proteins and enzymes related to larval immunity showed a significant decrease compared to that in *P. aeruginosa* strain single infections [80]. Relevant AMPs involved in antimicrobial activity, such as gloverin, cecropin D, and moricin, were clearly inhibited under coinfection conditions. All of them act against the bacterial cell wall by interacting with LPS and other components, especially affecting the outer membrane of gram-negative bacteria [81–83]. Specifically, moricin is capable of forming pores in gram-positive and gram-negative cell walls [84]. Under coinfection conditions, insect metalloproteinase inhibitor (IMPI) expression is also inhibited; this peptide has only been described thus far in *G. mellonella* [85] and inhibits the activity of proteases secreted by invading microorganisms [86]. In this sense, it is well known that *P. aeruginosa* produces various proteases as virulence factors [87]; thus, high levels of IMPI would potentially slow the progression of *P. aeruginosa* infection. Furthermore, IMPI inhibited the activation of phenoloxidase, an activator of the melanization cascade [88]. The melanin produced is deposited around the pathogenic microorganism, limiting its ability to damage the larva; however, parallel to melanin production, dangerous substances and reactive substances are also produced, resulting in tissue damage and cell death, which could be self-defeating for the host [89]. Additionally, the expression levels of immune-relevant proteins and enzymes, transferrin, lysozyme, and nitric oxide synthetase (NOS), are notably inhibited when *M. abscessus* and *P. aeruginosa* are infected simultaneously. Lysozyme is a cationic peptide with muramidase activity [90] that degrades peptidoglycan, making it more effective against gram-positive bacteria and, similar to IMPI, prevents an excessive melanization response [91]. Transferrin mediates nutritional immunity by sequestering iron from invading pathogens, thereby limiting their ability to grow and develop [92]. *P. aeruginosa* requires substantial amounts of iron to grow and produce two siderophores to take up this metal; therefore [93], high levels of transferrin in the hemolymph are beneficial to host survival. The production of nitric oxide by NOS under infection conditions is increased, although its role in immunity is not entirely clear [94]. It is hypothesized that it is transformed into reactive nitrogen species that are highly toxic to bacteria but equally toxic to host cells [95]. Moreover, the expression levels of glutathione S-transferase (GST) and apolipophorin III (Apo III) were clearly repressed only in *P. aeruginosa* PAO1-infected larvae, while they remained unchanged in other conditions, including *P. aeruginosa* PAET1, which indicates differences in the immune response against both *P. aeruginosa* strains. GST is a detoxification enzyme that protects hosts from oxidative stress [96]. To reduce pathogen progression, larvae induce a prophenoloxidase response, which triggers the production of reactive oxygen species that damage bacteria, although it also affects host cells [97], thereby inhibiting antioxidative enzymes, such as GST, under severe infections. Apo III is a lipid transport protein with relevant immune effects since it mediates hemocyte adhesion, phagocytosis, and nodule formation [98]. *P. aeruginosa* serine protease IV was shown to degrade Apo III [99,100]; however, the results of our study showed that Apo III levels were also reduced at the transcriptional level. In addition to the protease activity of *P. aeruginosa*, low levels of Apo III in the hemolymph can be reinforced by a repressive effect on its synthesis. NADPH oxidase (NOX4) is the only enzyme that does not show significant differences between infected and uninfected larvae. This enzyme produces superoxide and reactive oxygen species in response to infection, suggesting that it is not overexpressed under the infection conditions tested in this study because of the production of these toxic agents via the prophenoloxidase pathway [101], which is highly induced, as previously mentioned.

The repressive effect of *M. abscessus* R and S, together with *P. aeruginosa* PAO1 and PAET1 coinfections, was observed in most of the genes evaluated. However, as the only exception, the induction of transcriptional expression of the hemolin gene in *M. abscessus*-infected larvae (especially the R variant) was observed. Hemolin is an immunoglobulin-like protein with no direct antibacterial properties associated with hemocytes during the pathogen recognition process. This protein functions as an opsonin that mediates the interaction between bacteria and hemocytes and stimulates phagocytosis. Surprisingly, this study showed the phagocytic capacity of *G. mellonella* hemocytes against *M. abscessus* bacilli (Supplementary Figure S6), which could be directly related to the singular overexpression detected in mycobacterium-infected larvae.

Overall, the transcriptional expression levels of the different genes investigated showed high induction levels in the single infections of *P. aeruginosa*, although slight differences between the PAO1 and PAET1 strains were marked, showing different responses. In *M. abscessus*-infected larvae, expression levels were notably lower and remained at similar levels in both single and coinfections. This repression of the larval innate immune response could be attributed to the extensive phagocytosis of mycobacteria by hemocytes, which may proliferate and thereby limit the antibacterial activity of these cells, consequently hindering a robust specific response against *P. aeruginosa*. Additionally, previous studies have highlighted *M. abscessus* specific affinity of *M. abscessus* for colonizing and infecting the fat body tissue of *G. mellonella* larvae [102], which serves as the primary source of AMPs, enzymes, and antibacterial proteins secreted into the hemolymph [86]. Hence, the presence of *M. abscessus* in the fat body could account for the diminished production of these molecules, which correlates with the acceleration of lethality in the coinfection conditions. Notably, larvae infected with *B. thuringiensis* followed trends similar to those of individual *P. aeruginosa* infections, although interestingly showing lower levels.

The observed results of the immune response triggered by bronchial epithelial cells and *G. mellonella* larvae are compatible with the decreased response degree under coinfection and contrary to the overexpressed response under *P. aeruginosa* single infections. The hyperinflammatory response produced in the airways by *P. aeruginosa* infection is well described in patients [103], producing more severe respiratory malfunction that is usually treated with corticosteroids [104]. In the case of *M. abscessus*, it has been shown in a murine model of CF that the proinflammatory response produced is higher in the R morphotype than in the S morphotype [105], in accordance with the IL-6 and IL-8 differences detected in our study. The immune repression observed in the coinfections of *M. abscessus* and *P. aeruginosa* is described for the first time in this study, but further studies are needed to future research is needed to discern the molecular signaling pathways responsible for this effect. A disproportionate immune response is dangerous for the host because excess inflammation and the production of toxic compounds by bacteria and the host trigger a decline in survival. However, the weak immune response observed in coinfections with *M. abscessus* is even worse because the bacteria can spread unrestrictedly, triggering lethality at shorter times, as shown in the results of the *in vivo* survival and *in vitro* viability assays in this study. The findings addressed in the present study suggest the necessity of novel strategies to improve CF and other infection-related pulmonary disease management. New biofilm-disrupting methods, such as using enzymatic treatments, AMPs, or phage therapy in combination with antibiotics, are needed in order to improve efficacy and bacterial clearance. Additionally, testing novel immune modulator therapies can shift the immune balance from harmful inflammation to protective responses, potentially reducing tissue damage while improving infection control. Taking this study as a starting point and in order to generalize the results, it will be necessary to explore whether the results obtained are replicable using other strains of *P. aeruginosa* and *M. abscessus* beyond those employed in this study, particularly with clinical relevance, which allows the establishment of a clear pattern of interaction between both species when they are coinfecting simultaneously.

## Supplementary Material

Supplementary Table 2.docx

Supplementary Figure 42.jpg

Supplementary Figure 5C.tif

Supplementary Figure 6.tif

Supplementary Figure 41.jpg

Supplementary Figure 43.jpg

Supplementary Figure 2.jpeg

Supplementary Figure 5AB.tif

Supplementary Figure 1.jpg

QVIR-2024-0573.R1- clean copy of supplementary material.docx

Supplementary Figure 3.tif

Supplementary Table 1.docx

## Data Availability

The data generated during the study is available at repository name “Interplay of *Mycobacterium abscessus* and *Pseudomonas aeruginosa* in coinfection: Biofilm Dynamics and Host Immune Response” at https://doi.org/10.34810/data1720.
