# Supplementary material for: Interplay of Mycobacterium abscessus and Pseudomonas aeruginosa in experimental models of coinfection: Biofilm dynamics and host immune response
Source: Virulence. 2025 Apr 16;16(1):2493221. doi: 10.1080/21505594.2025.2493221 (PMC12064063; doi:10.1080/21505594.2025.2493221)
Supplement: Supplementary Table 2.docx [file KVIR_A_2493221_SM1055.docx]

| Primer name | Sequence 5´-3´ | Observations |
| --- | --- | --- |
| MbruPnrdHIE For | AAGGATCCGCGGTTCGCGACGCCGTC | Primers used to amplify the promoter region of the class Ib ribonucleotide reductase. This sequence was then used to clone into pFPV27 to obtain a constitutive expression of GFP (pETS218) |
| MbruPnrdHIE Rev | AAGGGCCCAGCGCCTTGTAGGTCGCGTT |  |
| 18S rRNA For | ATGGTTGCAAAGCTGAAACT | Housekeeping gene: Used to normalize gene expression data in RT-PCRs. |
| 18S rRNA Rev | TCCCGTGTTGAGTCAAATTA |  |
| Apo III For | AGACTTGCACGCCATCAAGA | Apolipophorin III: activity as pathogen recognition receptor, stimulating the activity of defense peptides, and possessing antimicrobial activity itself. |
| Apo III Rev | TGCATGCTGTTTGTCACTGC |  |
| Gloverin For | AGATGCACGGTCCTACAG | Interacts with lipopolysaccharides (LPS) and inhibit the formation of Gram-negative bacterial outer membrane. |
| Gloverin Rev | GATCGTAGGTGCCTTGTG |  |
| Lysozyme For | TCCCAACTCTTGACCGACGA | Is a muramidase that cleaves the linkages between N-acetylomuramid acid and N-acetylglucosamine in bacterial peptidoglycan. |
| Lysozyme Rev | AGTGGTTGCGCCATCCATAC |  |
| Cecropin D For | CTGCGCCATGTTCTTCA | Cationic antimicrobial peptide that disrupts microbial membranes, which eventually results in microbial cell death. |
| Cecropin D Rev | TCGCATCTCTGATCCTCTG |  |
| Moricin For | GCTGTACTCGCTGCACTGAT | Antibacterial activity against both Gram-positive and Gram-negative bacteria.Forms ion channels in the bacterial membrane. |
| Moricin Rev | TGGCGATCATTGCCCTCTTT |  |
| Hemolin For | CCCGAAGACGCTGGTGAATA | Functions as an opsonin that facilitates pathogen recognition and mediates hemocytic immune responses |
| Hemolin Rev | CGCACGTTCATTTGCTGTTC |  |
| GST For | GACAGAAGTCCTCCGGTCAG | Glutathione S-transferase: protect cells from oxidative stress, but they also play a central role in the detoxification of both endogenous and xenobiotic compounds. |
| GST Rev | TCCGTCTTCAAGCAAAGGCA |  |
| NOX-4 For | TGGCACGGCATCAGTTATCA | NADPH oxidase: is a pro-oxidative stress enzyme, induce oxidative stress humoral responses and secrete reactive oxygen species (ROS). |
| NOX-4 Rev | ACAGCGACTGTCATGTGGAA |  |
| NOS For | ATGAAGGTGCTGAAGTCACAA | Nitric oxide synthase: NO active the gene encoding of some antimicrobial peptides. |
| NOS Rev | GCCATTTTACAATCGCCACAA |  |
| IMPI For | ATTTGTAACGGTGGACACGA | Insect metalloproteinase inhibitor: protect AMPs against digestion by metalloproteinases secreted by the invading bacteria. |
| IMPI Rev | CGCAAATTGGTATGCATGG |  |
| Transferrin For | CCCGAAGATGAACGATCAC | Mediates nutritional immunity by sequestering iron from invading pathogens. Insects trigger a hypoferremic response after infection to limit iron availability to invading microbes. |
| Transferrin Rev | CGAAAGGCCTAGAACGTTTG |  |
