# Supplementary figures and images for: Interplay of Mycobacterium abscessus and Pseudomonas aeruginosa in experimental models of coinfection: Biofilm dynamics and host immune response
Source: Virulence. 2025 Apr 16;16(1):2493221. doi: 10.1080/21505594.2025.2493221 (PMC12064063; doi:10.1080/21505594.2025.2493221)

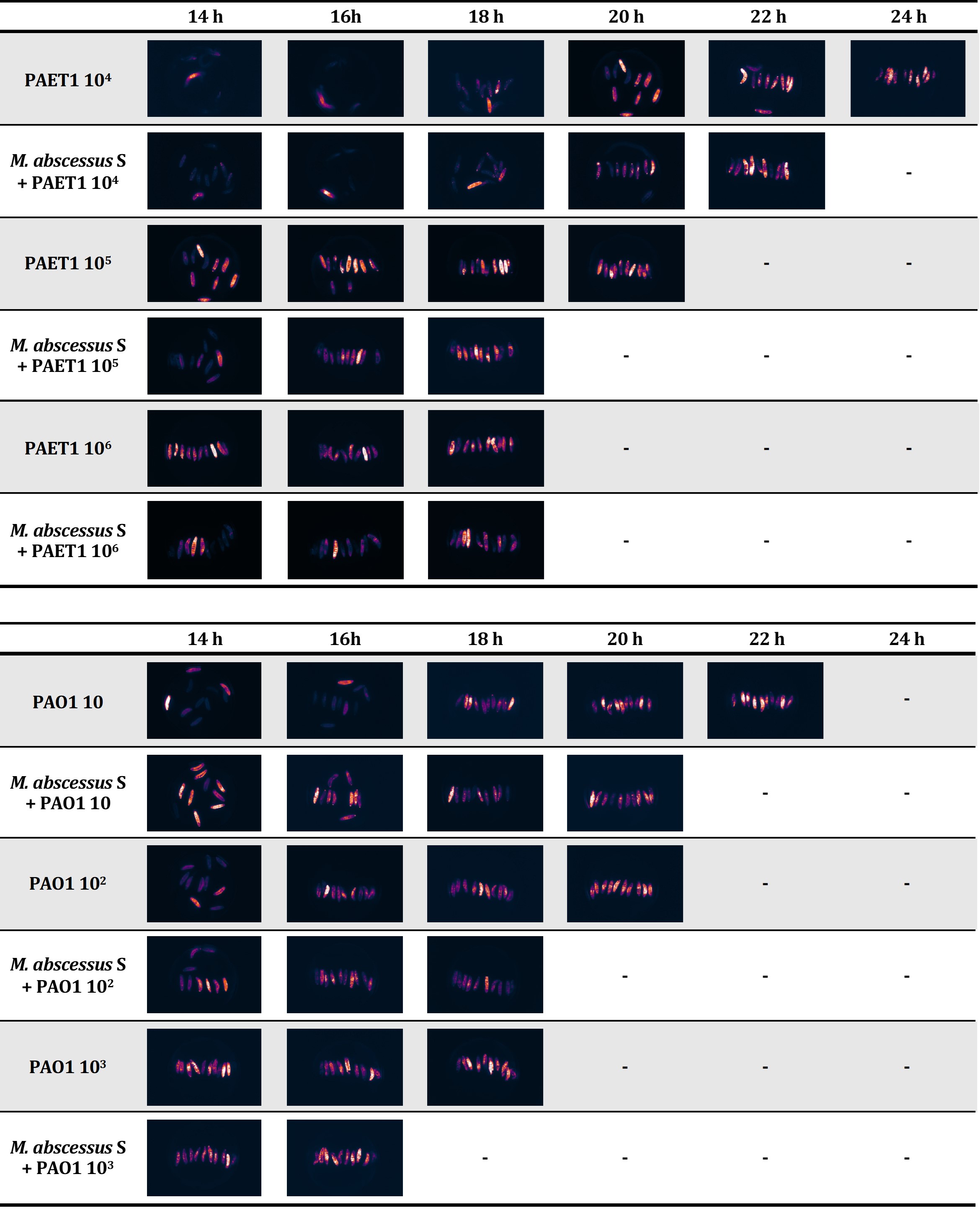

Supplement: Supplementary Figure 42.jpg [file KVIR_A_2493221_SM1054.jpg]

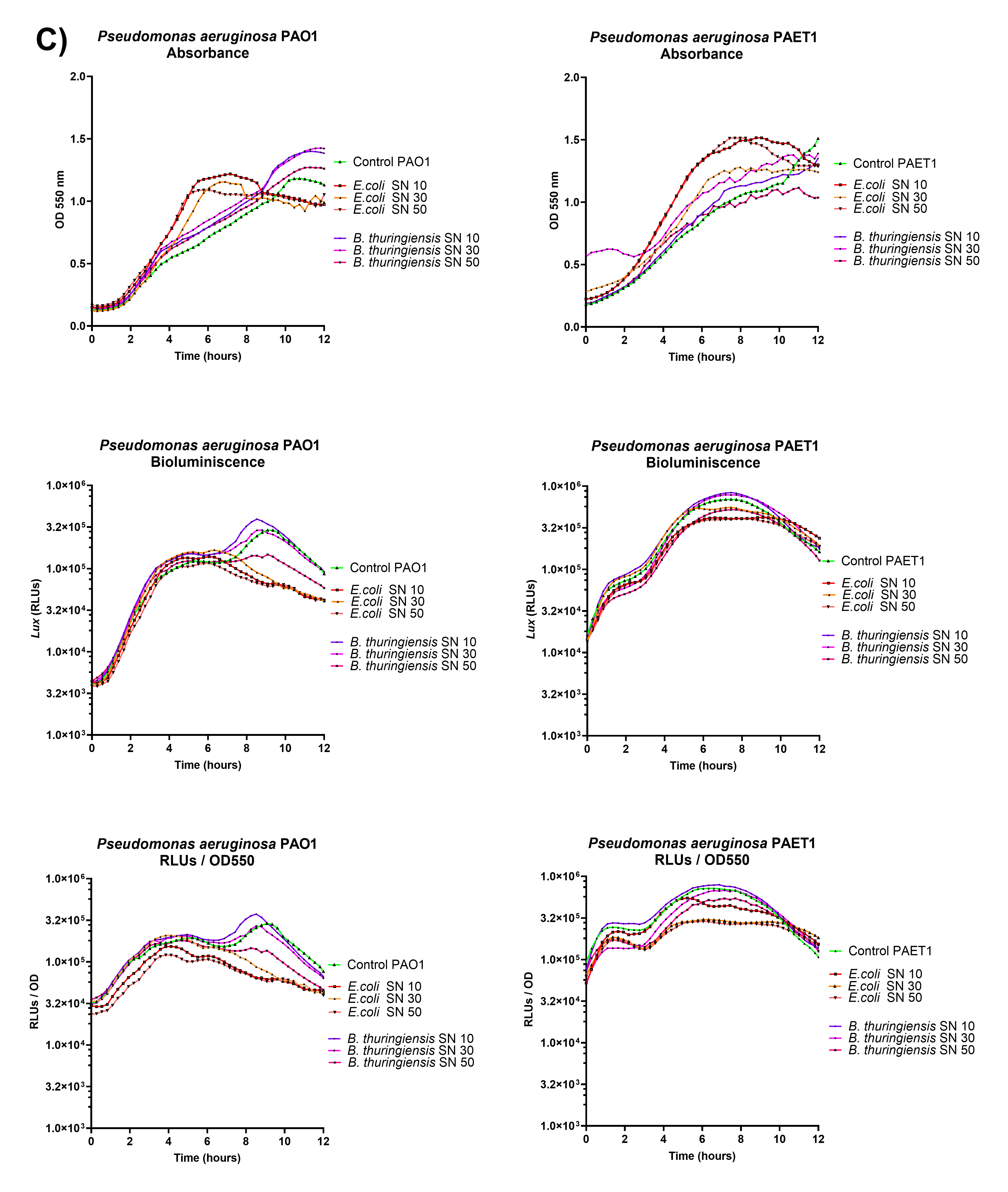

Supplement: Supplementary Figure 5C.tif [file KVIR_A_2493221_SM1053.tif]

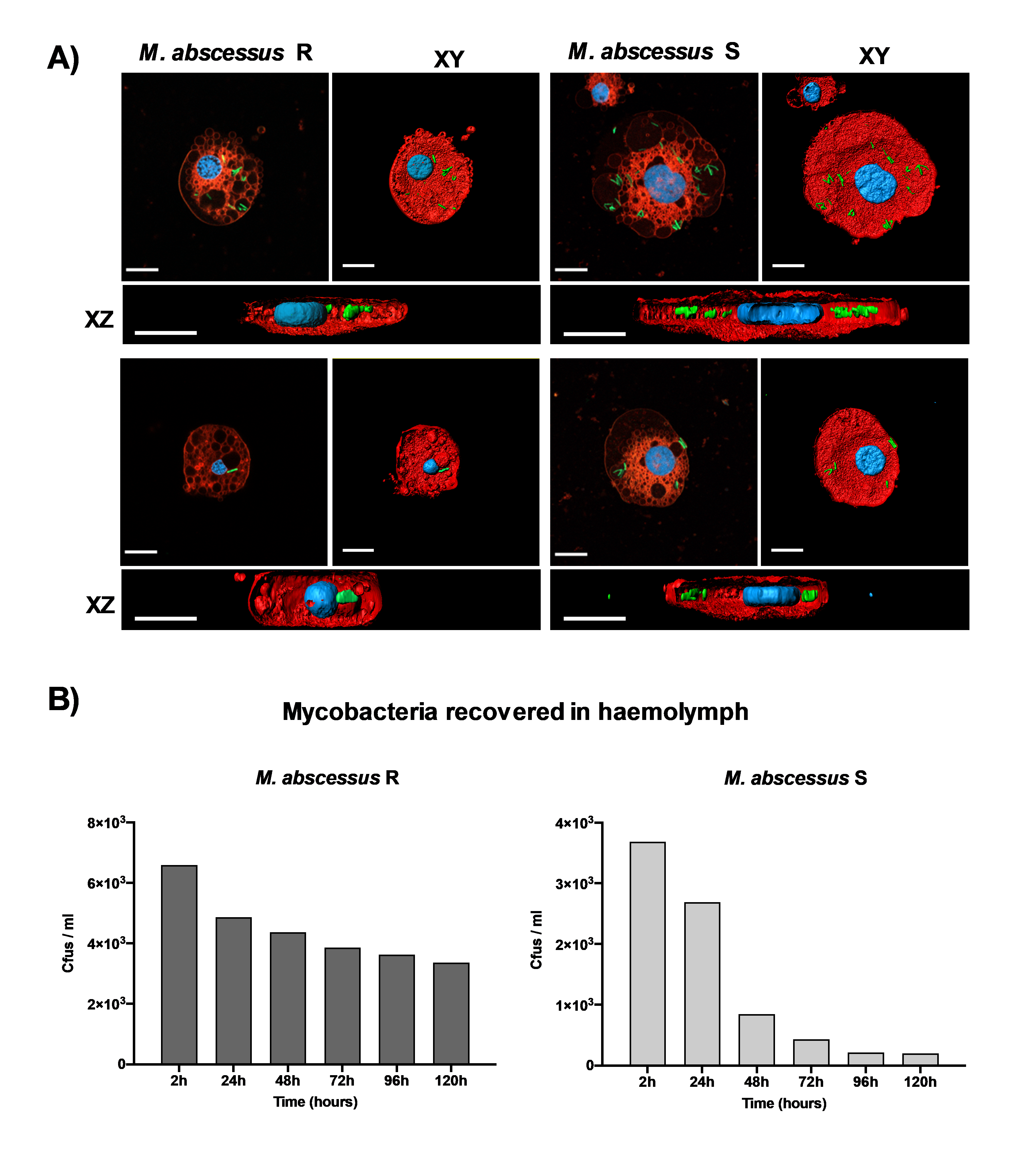

Supplement: Supplementary Figure 6.tif [file KVIR_A_2493221_SM1052.tif]

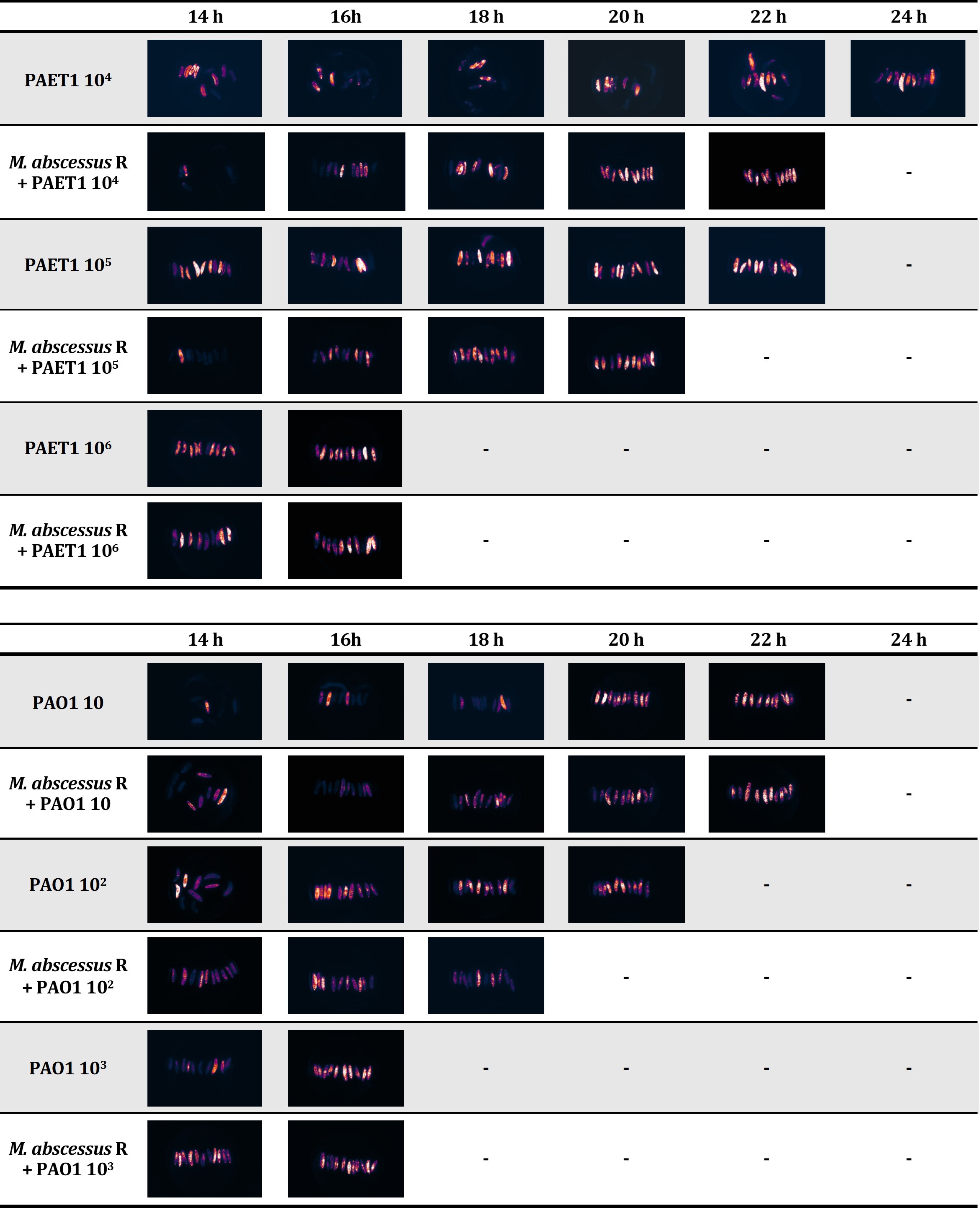

Supplement: Supplementary Figure 41.jpg [file KVIR_A_2493221_SM1051.jpg]

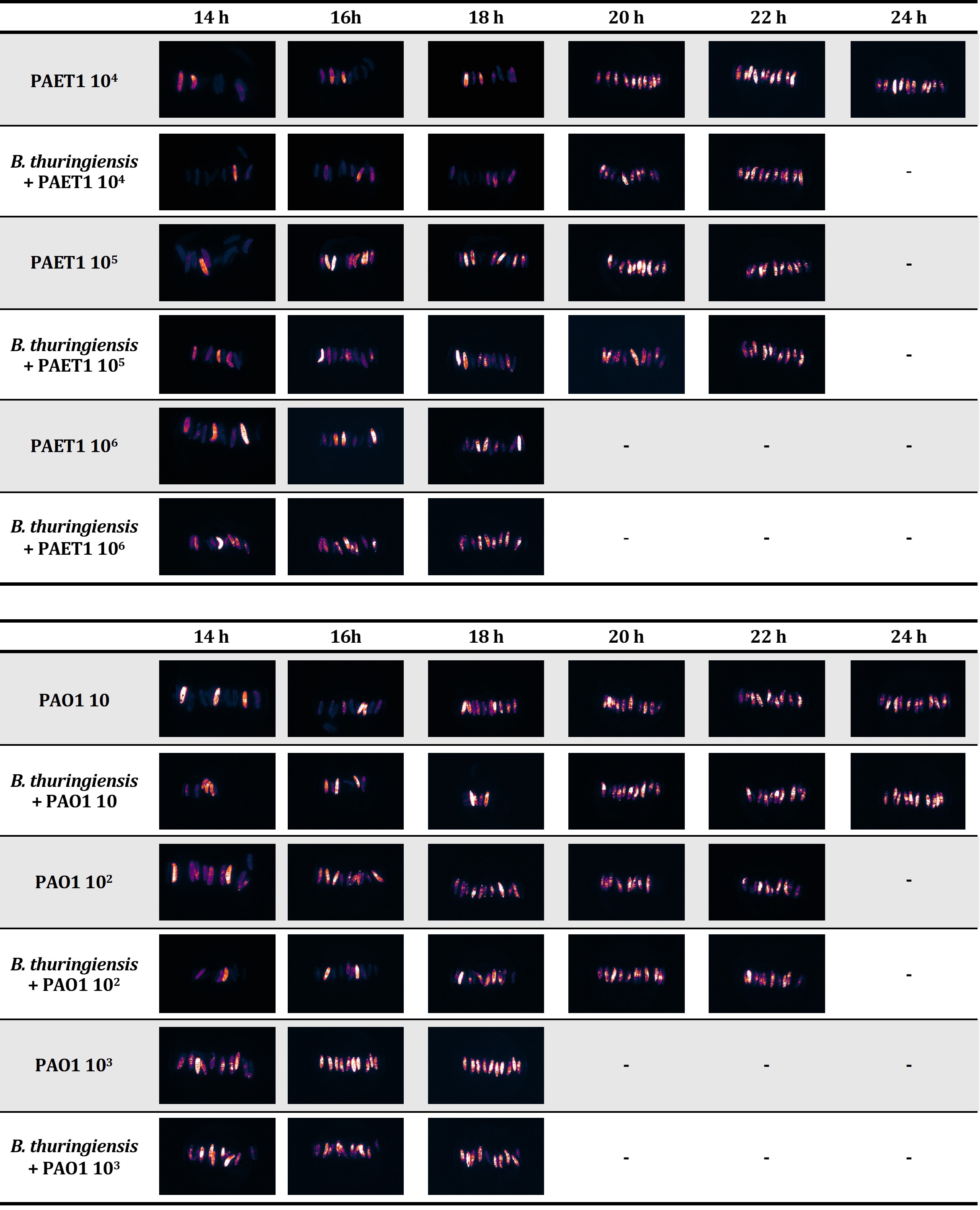

Supplement: Supplementary Figure 43.jpg [file KVIR_A_2493221_SM1050.jpg]

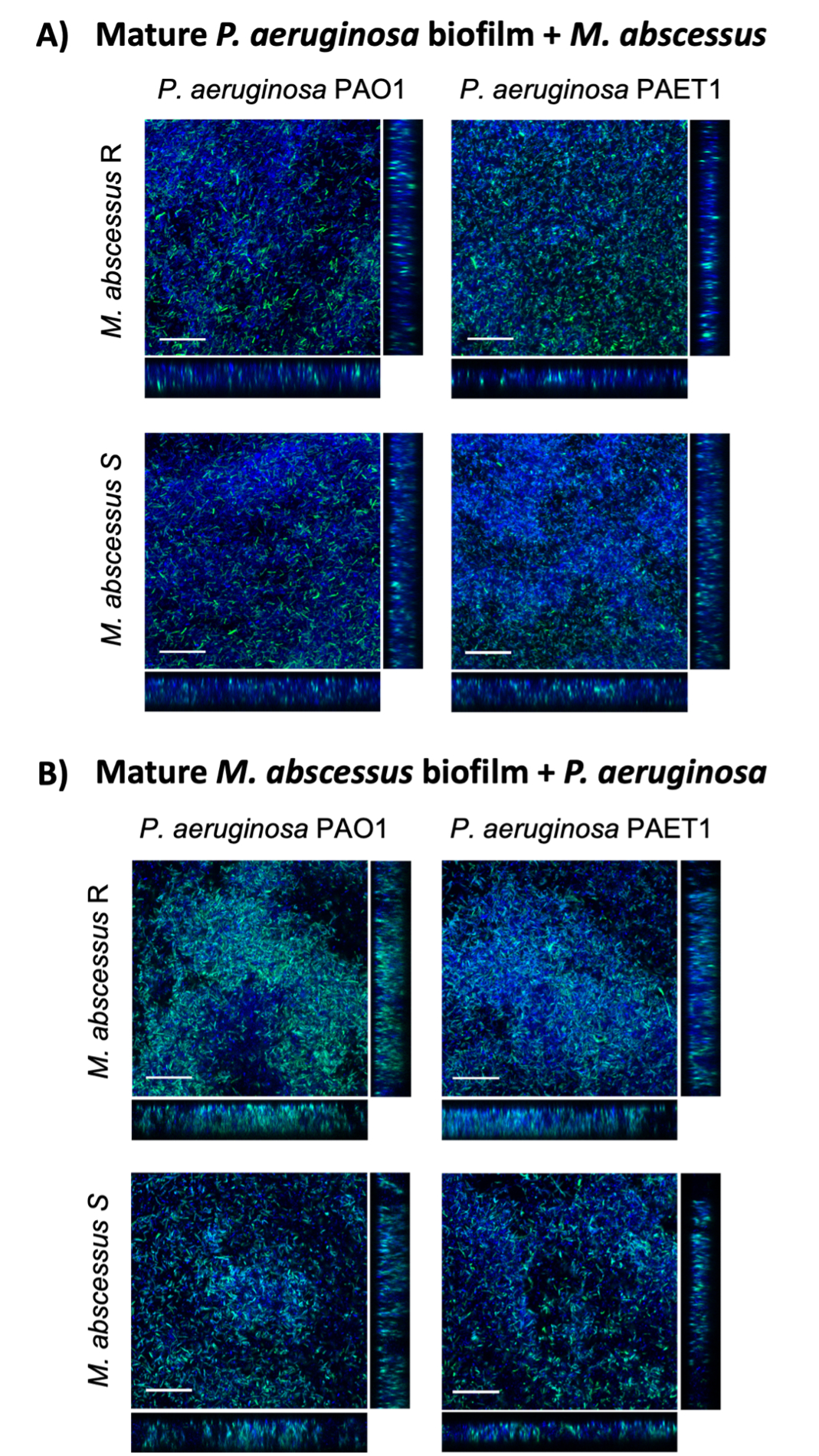

Supplement: Supplementary Figure 2.jpeg [file KVIR_A_2493221_SM1049.jpeg]

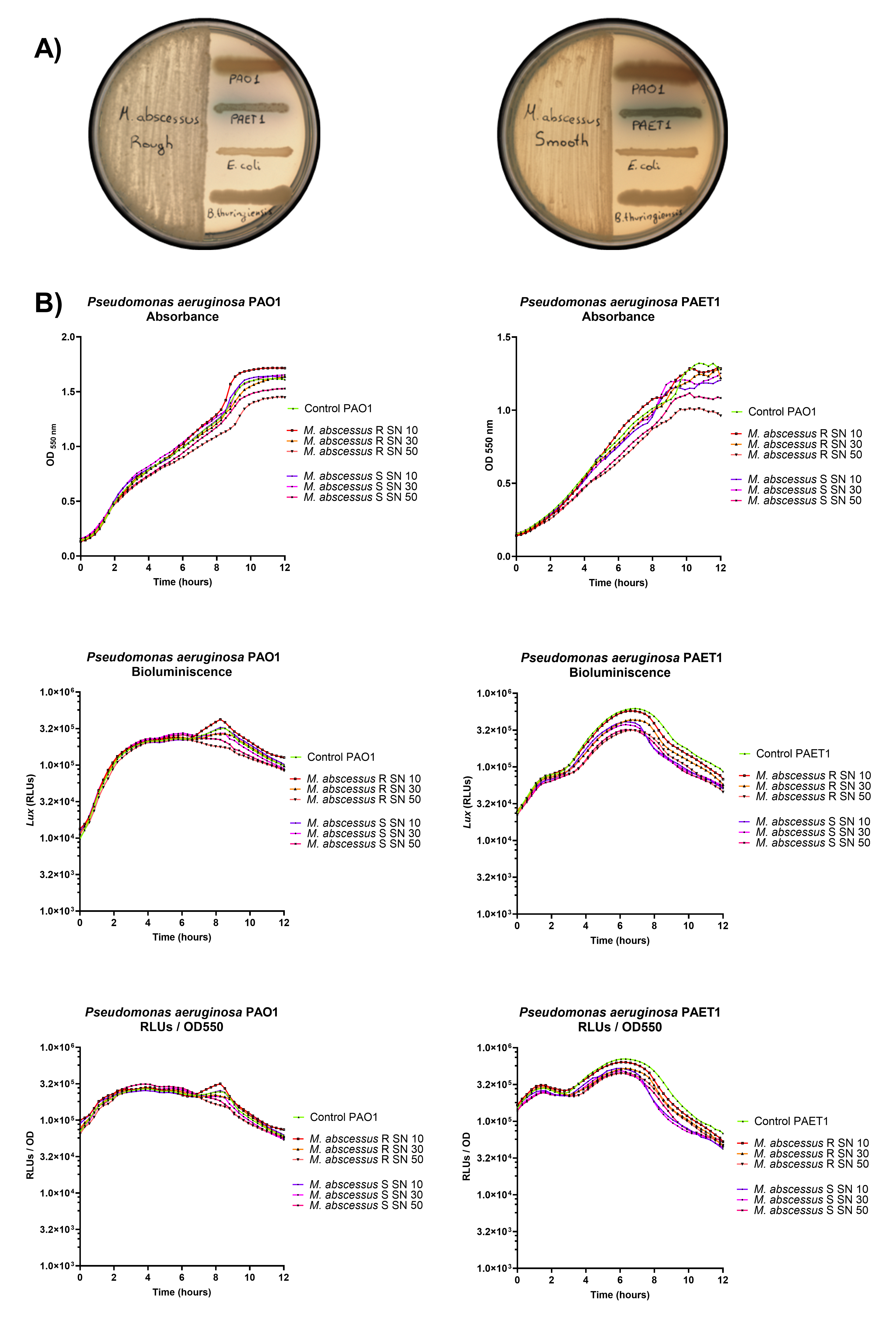

Supplement: Supplementary Figure 5AB.tif [file KVIR_A_2493221_SM1048.tif]

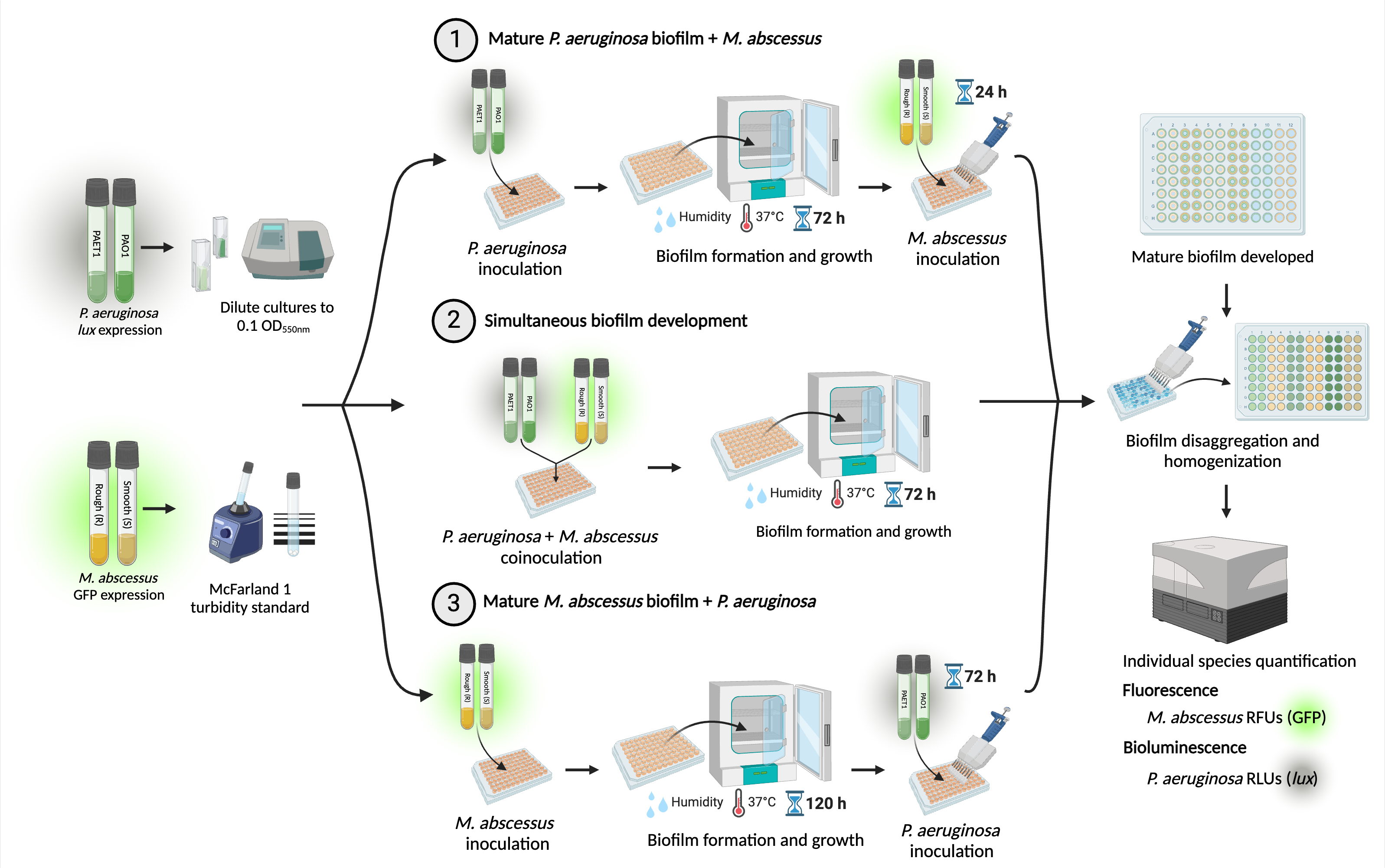

Supplement: Supplementary Figure 1.jpg [file KVIR_A_2493221_SM1047.jpg]

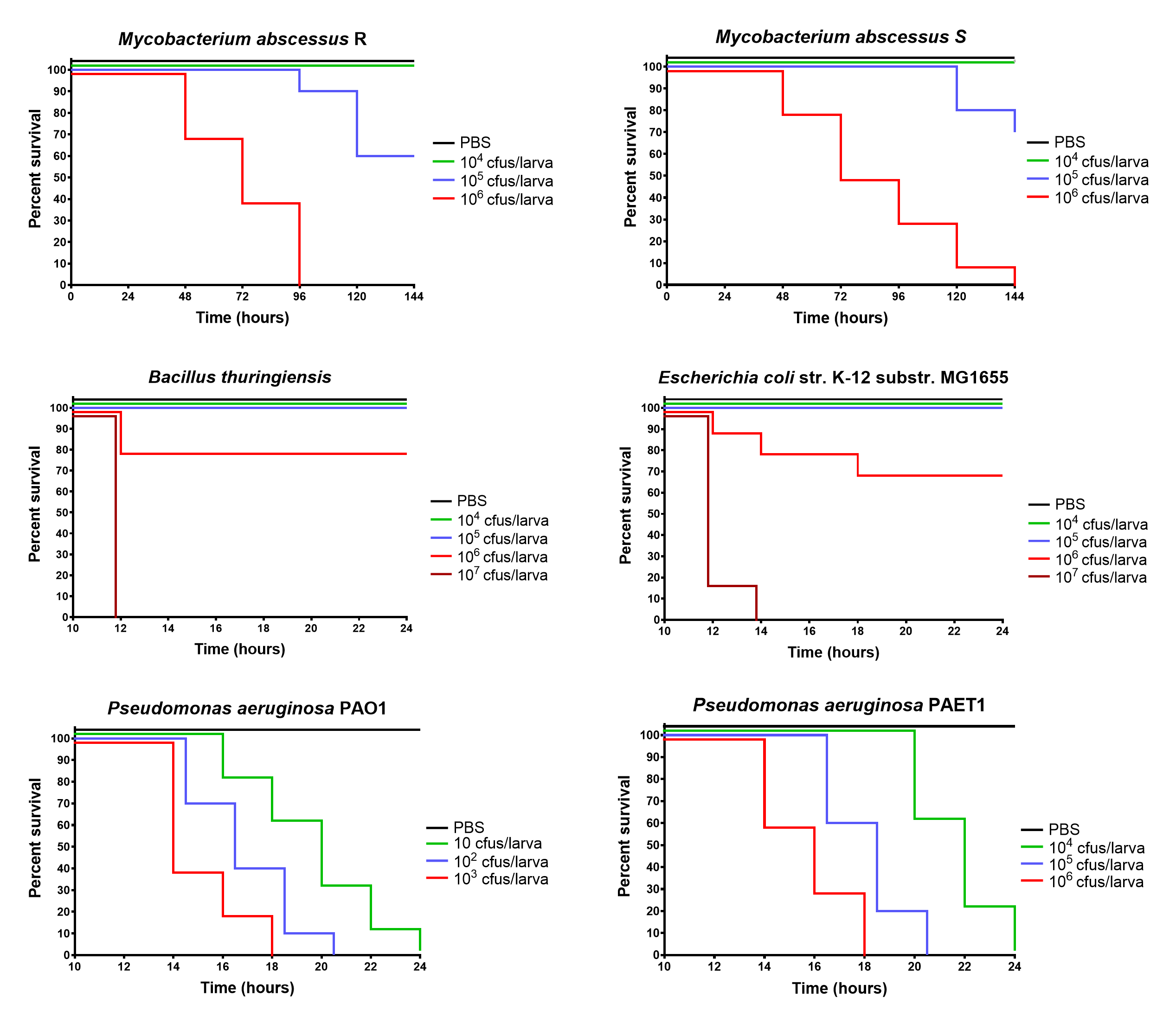

Supplement: Supplementary Figure 3.tif [file KVIR_A_2493221_SM1045.tif]
