# Supplementary material for: Interplay of Mycobacterium abscessus and Pseudomonas aeruginosa in experimental models of coinfection: Biofilm dynamics and host immune response
Source: Virulence. 2025 Apr 16;16(1):2493221. doi: 10.1080/21505594.2025.2493221 (PMC12064063; doi:10.1080/21505594.2025.2493221)
Supplement: Supplementary Table 1.docx [file KVIR_A_2493221_SM1044.docx]

| Strains | Observations |
| --- | --- |
| *Pseudomonas aeruginosa* PAO1 | Reference strain for laboratory studies. Compatible with a cystic fibrosis acute infection. |
| *Pseudomonas aeruginosa* PAET1 | Cystic fibrosis strain isolated from a chronic patient (88) |
| *Mycobacterium abscessus* Smooth morphotype | Type strain. (DSMZ 44916) |
| *Mycobacterium abscessus* Rough morphotype | Strain obtained by several passages of the original type strain (90) |
| *Escherichia coli* str. K-12 substr. MG1655 | Model strain in molecular biology. Safe strain used as control. |
| *Bacillus thuringiensis* | Type strain. (CECT 197) |
| *Escherichia coli* DH5α | Versatile strain used for general cloning applications. In the study it was used to preserve the different plasmids: pFPV27, pJET 1.2 + PnrdH, pETS218. All were transformed by thermal shook. |

| Plasmids | Observations |
| --- | --- |
| pFPV27 | Mycobacteria expression, it has a kanamycin resistance gene and another that codes for GFP. |
| pJET1.2 | Positive selection cloning vector |
| pETS130Lux | Plasmid previously constructed to obtain *Lux* constitutive expression in *P. aeruginosa* |
| pETS218 (MbruPnrdHIE + pFPV27) | Plasmid constructed to obtain GFP constitutive expression in *M. abscessus* |
